# Synthesis of nickel/gallium nanoalloys using a dual-source approach in 1-alkyl-3-methylimidazole ionic liquids

**DOI:** 10.3762/bjnano.10.171

**Published:** 2019-08-21

**Authors:** Ilka Simon, Julius Hornung, Juri Barthel, Jörg Thomas, Maik Finze, Roland A Fischer, Christoph Janiak

**Affiliations:** 1 Institut für Anorganische Chemie und Strukturchemie, Heinrich-Heine-Universität Düsseldorf, 40204 Düsseldorf, Germany; 2 Lehrstuhl für Anorganische und Metallorganische Chemie TU München, Lichtenbergstr. 4, 85748 Garching, Germany; 3 Gemeinschaftslabor für Elektronenmikroskopie RWTH-Aachen, Ernst Ruska-Centrum für Mikroskopie und Spektroskopie mit Elektronen, 52425 Jülich, Germany; 4 Department Structure and Nano-/Micromechanics of Materials, Max-Planck-Institut für Eisenforschung GmbH, 40237 Düsseldorf, Germany; 5 Institut für Anorganische Chemie, Institut für nachhaltige Chemie & Katalyse mit Bor (ICB), Julius-Maximilians-Universität Würzburg, Am Hubland, 97074 Würzburg, Germany

**Keywords:** ionic liquids, microwave decomposition, nickel/gallium nanoparticles, semihydrogenation catalysis, soft wet-chemical synthesis

## Abstract

NiGa is a catalyst for the semihydrogenation of alkynes. Here we show the influence of different dispersion times before microwave-induced decomposition of the precursors on the phase purity, as well as the influence of the time of microwave-induced decomposition on the crystallinity of the NiGa nanoparticles. Microwave-induced co-decomposition of all-hydrocarbon precursors [Ni(COD)_2_] (COD = 1,5-cyclooctadiene) and GaCp* (Cp* = pentamethylcyclopentadienyl) in the ionic liquid [BMIm][NTf_2_] selectively yields small intermetallic Ni/Ga nanocrystals of 5 ± 1 nm as derived from transmission electron microscopy (TEM) and high-angle annular dark-field scanning transmission electron microscopy (HAADF-STEM) and supported by energy-dispersive X-ray spectrometry (EDX), selected-area energy diffraction (SAED) and X-ray photoelectron spectroscopy (XPS). NiGa@[BMIm][NTf_2_] catalyze the semihydrogenation of 4-octyne to 4-octene with 100% selectivity towards (*E*)-4-octene over five runs, but with poor conversion values. IL-free, precipitated NiGa nanoparticles achieve conversion values of over 90% and selectivity of 100% towards alkene over three runs.

## Introduction

The synthesis of Ni nanoparticles is well known and is most commonly carried out in organic solvents using reducing agents [1], thermal decomposition [2] or reductive hydrogenation [3]. Applications for Ni nanoparticles are Wittig-type olefination [4], Suzuki cross-coupling [5] and catalytic hydrogenation reactions [6]. The catalytic activity of Ni nanoparticles can be used in hydrogenation reactions of alkenes [7], styrene [8], and quinoline [9]. Semihydrogenation reactions of alkynes lead to overhydrogenation [10] or polymerization in the case of acetylene to form oligomers [11]. One can distinguish between the more stable face-centered cubic (fcc) [12] and the less stable hexagonal close-packed (hcp) [13] Ni phase. The magnetic properties of fcc Ni nanoparticles are similar to the bulk material with saturation magnetization values of 50 emu/g_Ni_ at 300 K [14]. Hcp Ni nanoparticles show very weak magnetic features with saturation magnetization values below 1 emu/g_Ni_ at 300 K [15]. Ni nanoparticles can easily be prepared from bis(1,5-cyclooctadiene)nickel(0) (Ni(COD)_2_) in organic solvents [16] with the Ni atom already in the oxidation state zero and a low decomposition temperature of 60 °C [17]. Alternatively, ionic liquids can be used as solvents and stabilization agents for different metal nanoparticles from metal carbonyls [18] or organometallic complexes [19]. Ni nanoparticles from Ni(COD)_2_ in ionic liquids can be obtained through spontaneous decomposition [20] or decomposition induced by microwave heating [21] as well as through ligand hydrogenation [22].

The complete removal of alkynes from alkenes is very important in industrial olefin polymerization reactions. Examples are the separation of acetylene from ethylene [22–23] or of phenylacetylene from styrene [24]. The presence of small quantities of alkynes significantly reduces the efficiency of catalysts in the subsequent polymerization reactions. Semihydrogenation reactions are an interesting way not only to remove but also to convert the alkynes to the respective polymerizable alkenes [25]. The addition of main-group metals such as gallium to transition metals can significantly improve the catalytic selectivity towards semihydrogenation reactions, e.g., PdGa [26–28] and RhGa [29]. Intermetallic nanoparticles of nickel and gallium have been proven as efficient catalysts in semihydrogenation reactions experimentally [30–31] and reasoned by theory [32].

The phase diagram of Ni/Ga shows nine different Ni/Ga phases (Supporting Information File 1, Figure S1) [33–36]. In a comparison of the CO_2_ hydrogenation abilities of NiGa (β), Ni_3_Ga (α) and Ni_5_Ga_3_ (δ) high selectivities towards the formation of methanol were found for Ni_5_Ga_3_ and NiGa [37]. At 165 °C Ni_5_Ga_3_ (δ) yielded 100% selectivity towards methanol [38]. Above 220 °C Ni_5_Ga_3_ is even more active than a conventional Cu/ZnO/Al_2_O_3_ catalyst with less CO formation in the reverse water-gas shift reaction (rWGS). In Ni_5_Ga_3_ the Ga-rich step sites facilitate the methanol synthesis, the Ni-rich sites get self-poisoned by methanation and CO formation through rWGS [37]. In conclusion, Ni_5_Ga_3_(δ) was found to be the most active catalyst for CO_2_ hydrogenation [39–41]. Semihydrogenation of phenylacetylene to styrene using NiGa, Ni_3_Ga and Ni_5_Ga_3_ as catalysts indicated that using Ni_3_Ga (α) yielded the highest activity with a turnover frequency (TOF) of 5.16 × 10^−3^ h^−1^ with the highest selectivities [10,30,32,42].

Bimetallic nanoparticles containing Ga are difficult to synthesize from Ga^3+^ precursors, because of the high negative redox potential of Ga^3+^ as well as the low melting point of Ga metal leading to coagulation. During the synthesis of NiGa [42] or PdGa [27] nanoparticles from Ni^2+^ or Pd^2+^ precursors, using aminoborane as reducing agent, the formation of a transition-metal hydride was reported as a first step. These hydrides can then reduce the Ga^3+^ precursor, working as a nucleation center for the Ga atoms and prevent uncontrolled coagulation of the liquid metal. Through annealing, the single-phase products can be obtained [27,42].

The all-hydrocarbon precursor GaCp* (Cp* = pentamethylcyclopentadienyl), with the Ga atom in the oxidation state +1, was reported to form phase-pure NiGa and Ni_3_Ga nanoparticles with Ni(COD)_2_ in the ionic liquid [BMIm][BF_4_] under microwave-induced pyrolysis at 230 °C [30]. GaCp* is reported to be thermally stable in organic solvents in the absence of hydrogen to up to 300 °C [43]. In imidazolium-based ionic liquids decomposition of GaCp* is possible at temperatures below 300 °C with the aid of transition metals. Reactions of transition-metal complexes are reported to show H/D activation/exchange reactions at the C2 imidazolium carbon atom of the ionic liquid cation. The generated N-heterocyclic carbene ligands (NHC) stabilize metal clusters and nanoparticles [44]. By insertion of the transition-metal center into the C2–H bond of imidazolium salts, transition-metal hydride complexes are formed [45]. Finally, H transfer reactions from the transition metal to GaCp* lead to the release of Cp*H without additional hydrogen [46]. Here, small NiGa nanoparticles were synthesized from Ni(COD)_2_ and GaCp* in the ionic liquid [BMIm][NTf_2_]. The nanoparticles were characterized and tested for the semihydrogenation reaction of 4-octyne following our work on the selective semihydrogenation reaction of the terminal alkyne 1-octyne and the internal alkyne diphenylacetylene with yields of 90% and selectivities of 94% and 87%, respectively [30].

## Results and Discussion

**Figure 1:**
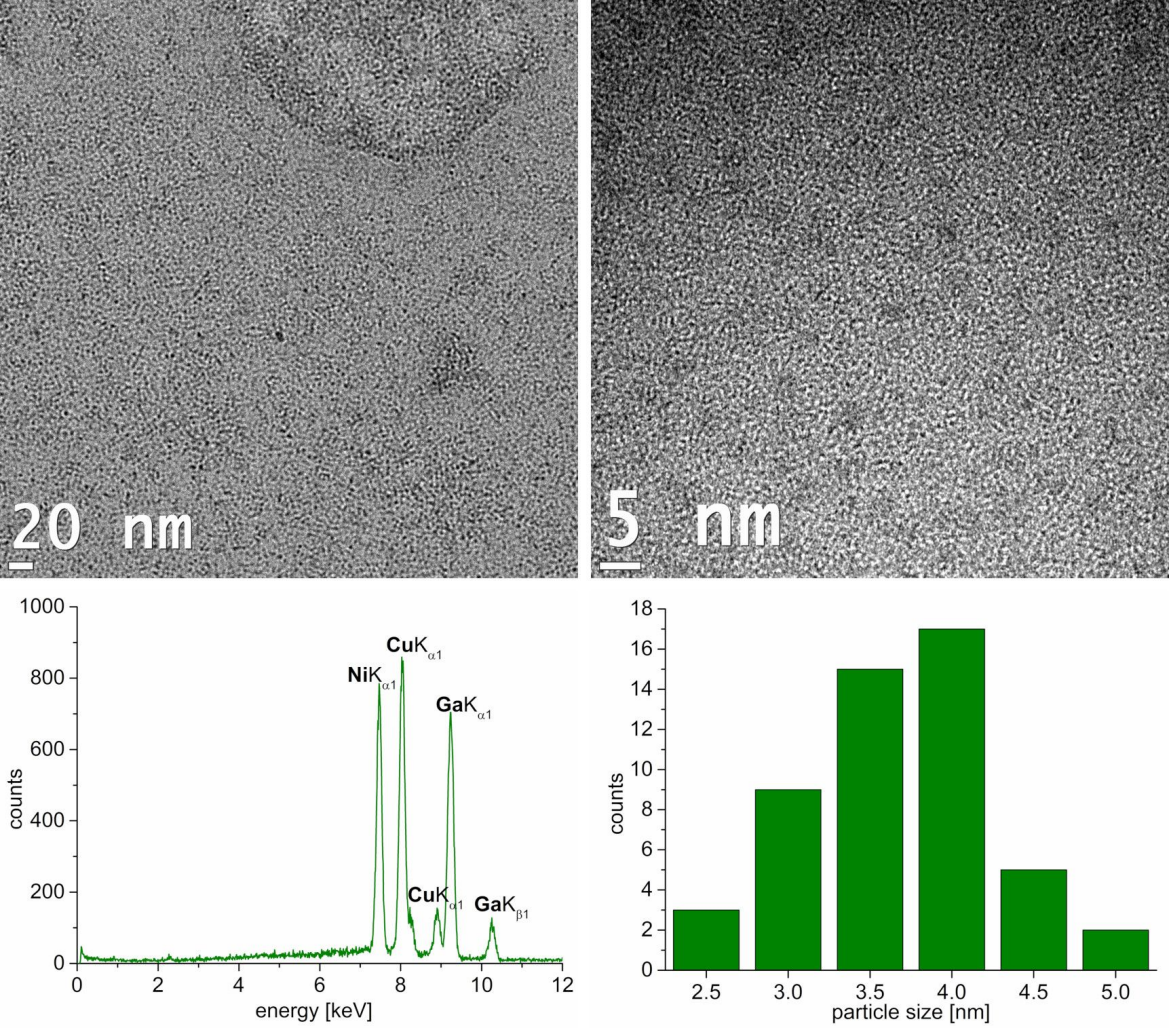
Top: HRTEM images of Ni/Ga nanoparticles from a 0.5 wt % dispersion of Ni(COD)_2_ and GaCp* in [BMIm][NTf_2_] after 24 h of dispersion and 10 min of microwave-induced decomposition. Bottom: EDX spectrum and particle size distribution 3 ± 0.5 nm (87 particles counted).

Ni(COD)_2_ and GaCp* were dispersed in equimolar ratio in [BMIm][NTf_2_] for 24 h prior to the thermal decomposition. Through microwave irradiation at 230 °C, a black powder was obtained after 10 min. The TEM measurements show spherical and non-aggregated nanoparticles with a narrow size distribution of 3.0 ± 0.5 nm (Figure 1). To validate the intermetallic 1:1 NiGa phase of the obtained nanoparticles, powder X-ray diffraction pattern (P-XRD) or selected-area energy diffraction (SAED) are required. Presumably, due to the small size of the nanoparticles, these measurements yielded no diffractograms. Therefore, the nanoparticles can only be described as non-crystalline or amorphous. Quantification of EDX spectra from three different spots on the TEM grid gave a nearly equimolar ratio of nickel to gallium of 46:54 atom % (±1 atom %). No oxygen peak was detected.

**Figure 2:**
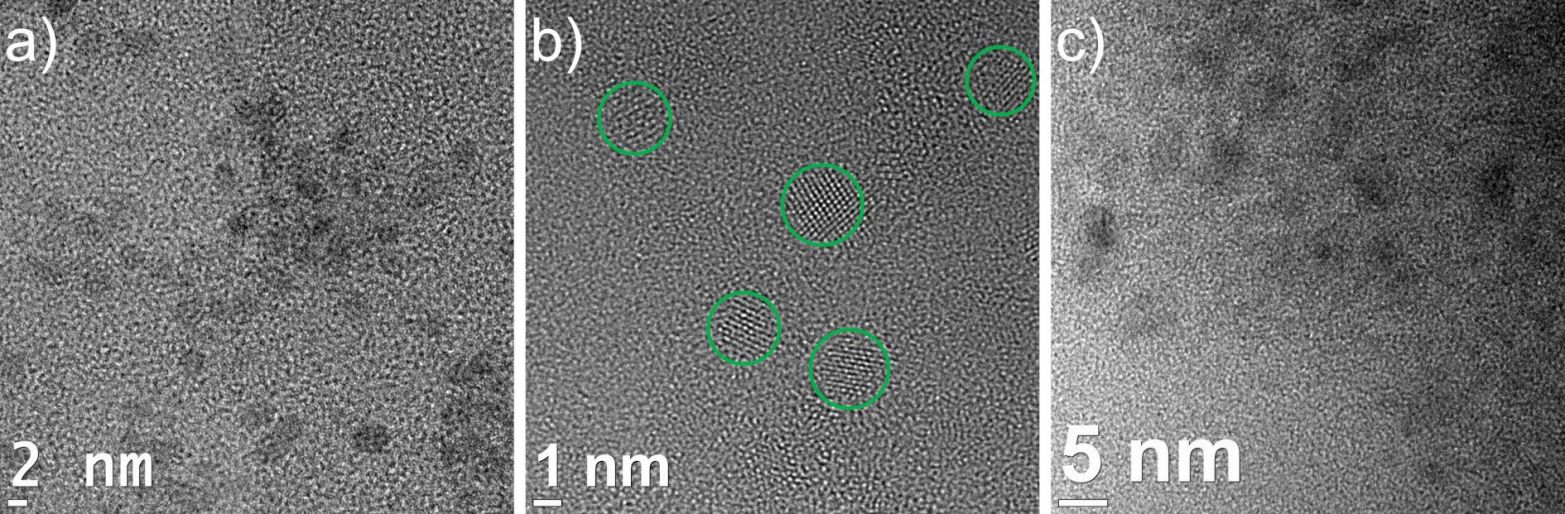
HRTEM images: a) Ni/Ga nanoparticles from a 1 wt % dispersion of Ni(COD)_2_ and GaCp* in [BMIm][BF_4_] after 24 h of dispersion and 10 min of microwave-induced decomposition. For details of the particle size distribution (2.5 ± 0.5 nm), see Supporting Information File 1, Figure S2. b) Ni/Ga nanoparticles from 0.5 wt % dispersion of Ni(COD)_2_ and GaCp* in [BMIm][BF_4_] after 24 h of dispersion and 20 min of microwave-induced decomposition. For details of the particle size distribution (2.5 ± 0.5 nm), see Supporting Information File 1, Figure S3. c) Ni/Ga nanoparticles from 0.5 wt % dispersion of Ni(COD)_2_ and GaCp* in propylene carbonate after 24 h of dispersion and 20 min of microwave-induced decomposition. For details of the particle size distribution (5 ± 1 nm), see Supporting Information File 1, Figure S4.

The ionic liquid [BMIm][BF_4_] and the organic propylene carbonate (PC) yield, under the same reaction conditions, small non-aggregated and non-crystalline Ni/Ga nanoparticles (Figure 2) of a size distribution of 2.5 ± 0.5 nm ([BMIm][BF_4_], Supporting Information File 1, Figure S2 and Figure S3) and 5 ± 1 nm (PC, Supporting Information File 1, Figure S4). EDX quantification over different spots on the TEM grid also shows equimolar ratios of nickel to gallium (Supporting Information File 1, Figure S2 and Figure S3).

**Figure 3:**
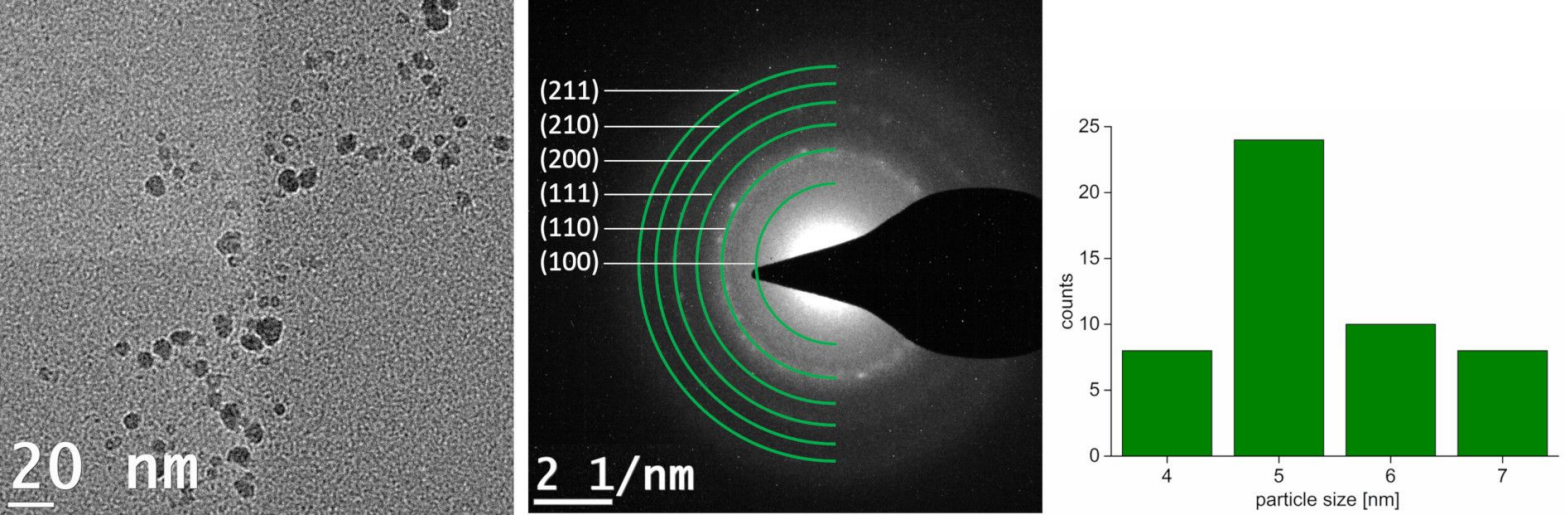
HRTEM image and particle size distribution of 0.5 wt % dispersion of NiGa nanoparticles from Ni(COD)_2_ and GaCp* in [BMIm][NTf_2_] after 24 h of dispersion and 30 min of microwave-induced decomposition. SAED with indexed reflections for NiGa (space group 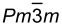). Particle size distribution 5 ± 1 nm (50 particles counted).

Annealing of nanoparticle samples is known to improve the crystallinity of the nanoparticles [47–48]. The decomposition of Ni(COD)_2_ and GaCp* in [BMIm][NTf_2_] was repeated under the same conditions, but with a longer decomposition time of 30 min, in order to induce annealing in the microwave reactor. Subsequently, the TEM images show spherical and crystalline nanoparticles with a small size distribution of 5 ± 1 nm (Figure 3). Through the increased decomposition time the particles were grown slightly larger. The metal composition quantification by EDX spectra from three different spots on the TEM grid gave an equimolar ratio of nickel to gallium of 47:53 atom % (±1 atom %). The formation of intermetallic NiGa (β) nanoparticles was verified by SAED measurements (Figure 3, NiGa space group 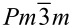).

**Figure 4:**
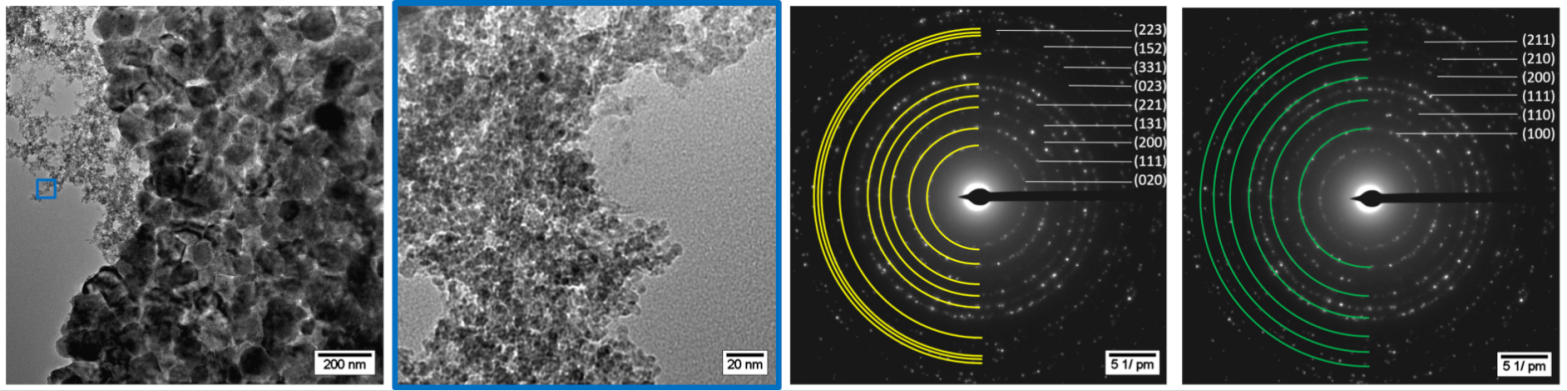
HRTEM images of NiGa nanoparticles (small particles) and Ga(Ni) nanoparticles (large particles) from a 0.5 wt % dispersion of Ni(COD)_2_ and GaCp* in [BMIm][NTf_2_] after 1 h of dispersion prior to 30 min of decomposition. SAED with indexed reflections for Ga (yellow diffraction rings for space group *Cmce*) and NiGa (green diffraction rings for space group 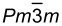).

To elucidate the influence of the dispersion time prior to the microwave-induced thermal decomposition of Ni(COD)_2_ and GaCp*, two samples with shorter dispersion times of 1 h and 12 h were prepared. Ni(COD)_2_ and GaCp* were dispersed in equimolar ratio in [BMIm][NTf_2_] for 1 h prior to the thermal decomposition. Through microwave irradiation at 230 °C, a black powder was obtained after 30 min. The TEM measurements show two different sizes of spherical, crystalline and aggregated nanoparticles (Figure 4). The SAED patterns can be differentiated into the cubic NiGa phase and orthorhombic Ga(Ni) phase (Figure 4, space group: NiGa 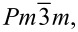 Ga(Ni): *Cmce.* For the SAED pattern of only the small particles see Supporting Information File 1, Figure S5, bottom). The presence of the Ga-rich phases Ni_3_Ga_4_, Ni_2_Ga_3_, Ni_3_Ga_7_ and NiGa_5_, which exist in the Ni/Ga phase diagram can be excluded by SAED measurements (for comparison see Supporting Information File 1, Table S1).

Quantification of the EDX spectrum of mostly the large particles show average ratios of nickel to gallium of 28:72 atom % (±2 atom %, Supporting Information File 1, Figure S5 top left, for individual values see Supporting Information File 1, Table S2). The missing nickel from the initial equimolar ratio can be explained by the possible formation of NiCp*, which is stable up to 290 °C [49]. Quantification of the EDX spectrum of only the small particles shows an equimolar ratio of nickel to gallium of 52:48 atom % (±2 atom %) (Supporting Information File 1, Figure S5, top right). Therefore, the small nanoparticles with a size distribution of 6 ± 1 nm can be assigned to be NiGa nanoparticles. Moreover, the large particles with a size distribution of 90 ± 20 nm can be assigned to be Ga-rich nanoparticles. We suggest that they cannot be pure Ga nanoparticles, because the low melting point of Ga metal of 30 °C, would yield liquid Ga metal under the energy of the electron beam in the TEM. Thus, the orthorhombic Ga phase probably contains a few percent of metallic nickel.

**Figure 5:**
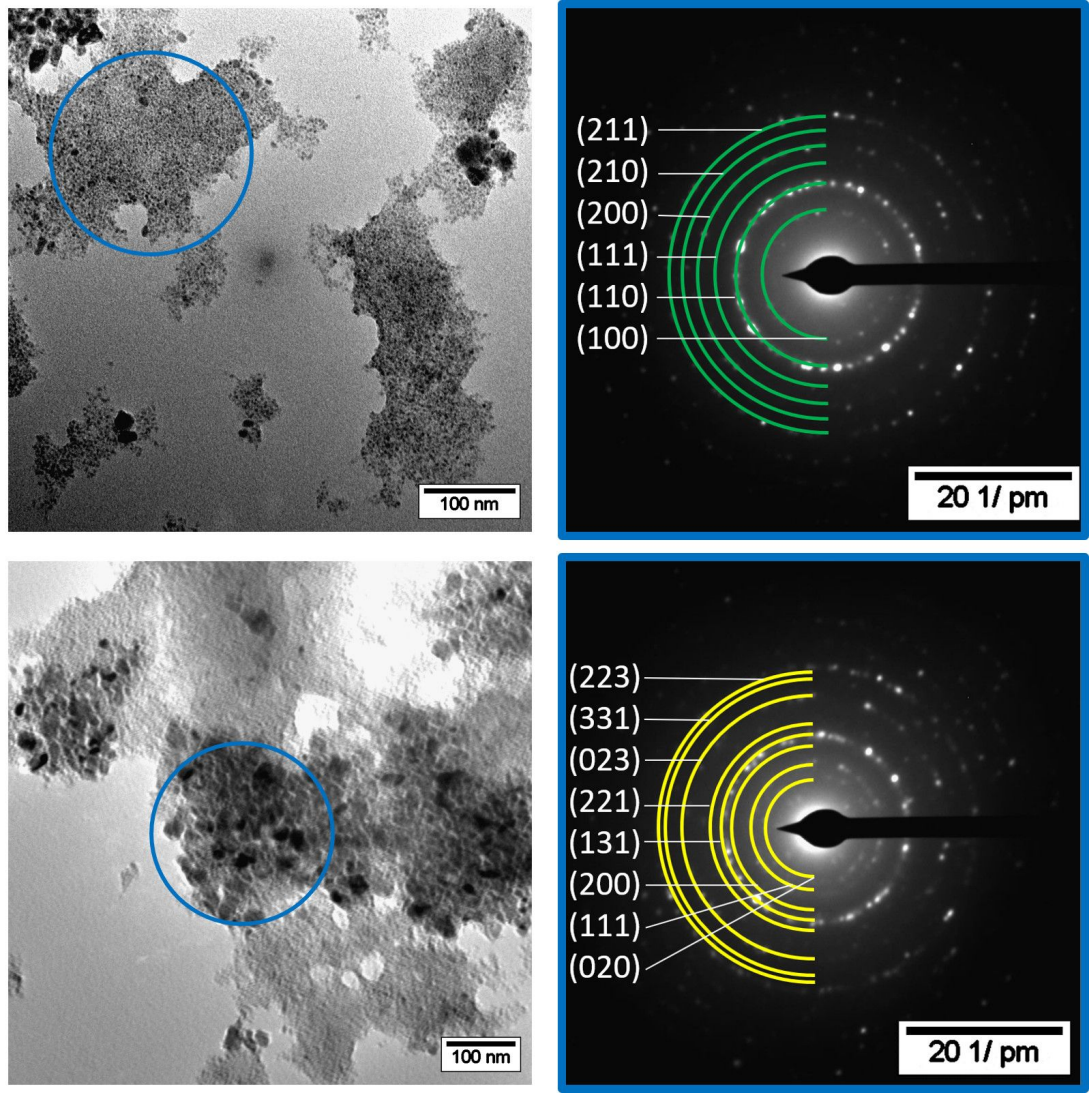
HRTEM images of NiGa nanoparticles (small particles) and Ga(Ni) nanoparticles (large particles) from a 1 wt % dispersion of Ni(COD)_2_ and GaCp* in [BMIm][NTf_2_] after 12 h of dispersion and 30 min of decomposition. SAED with indexed reflections for NiGa (green diffraction rings for space group 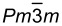) and Ga (yellow diffraction rings for space group *Cmce*).

Similarly, Ni(COD)_2_ and GaCp* were dispersed in equimolar ratio in [BMIm][NTf_2_] for 12 h prior to the thermal decomposition. Through microwave irradiation at 230 °C, a black powder was obtained after 30 min. The TEM measurements show spherical, crystalline and aggregated small nanoparticles with size distribution of 7 ± 1 nm and large particles with a size distribution of 30 ± 10 nm (Figure 5). As before, the corresponding SAED patterns show cubic NiGa for the small particles and orthorhombic Ga(Ni) for the large particles (Figure 5, space group: NiGa 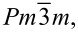 Ga: *Cmce*). The quantification of EDX spectra from three different spots on the TEM grid shows an averaged ratio of nickel to gallium of 38:62 atom % (±1 atom %) (for individual values see Supporting Information File 1, Table S2).

**Figure 6:**
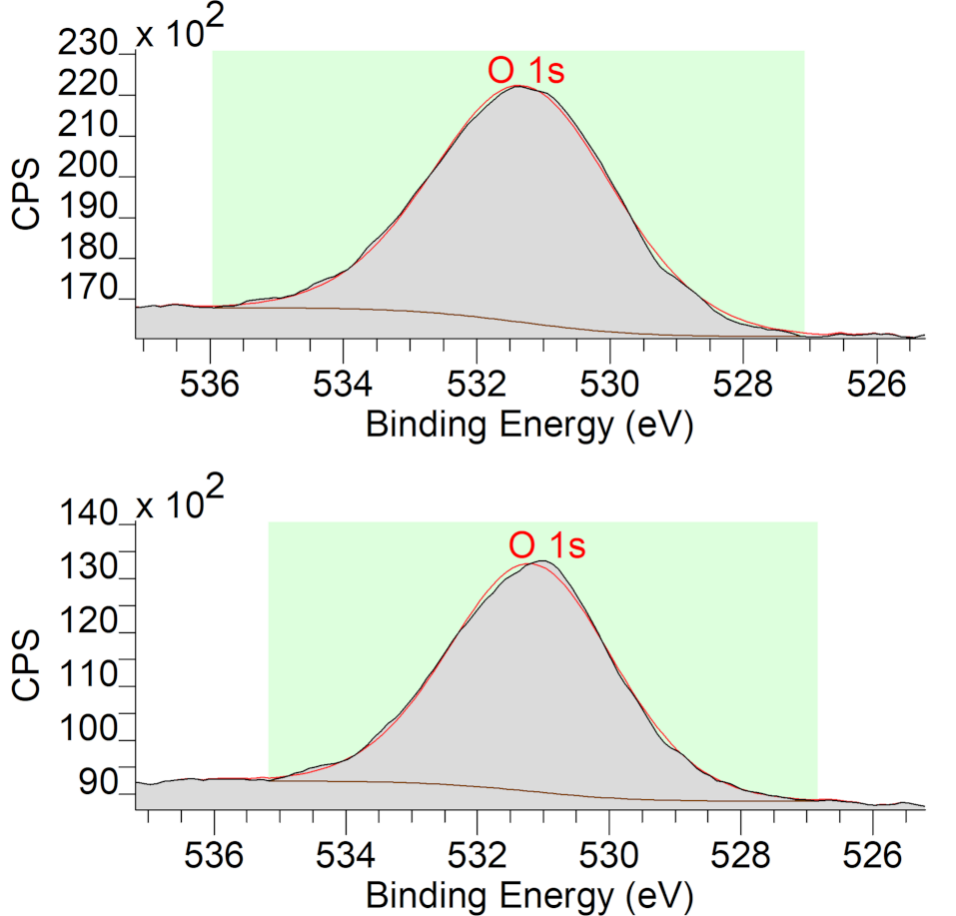
HRXPS region of the O 1s orbital of NiGa/Ga(Ni) nanoparticle samples obtained by microwave-induced decomposition after 1 h of dispersion time (top) and after 12 h of dispersion (bottom).

By using high-resolution X-ray photoelectron spectroscopy (HRXPS), the electron binding energy of the O 1s orbital was measured to confirm that the Ga nanoparticles in both samples are doped with NiGa and not with Ga oxide (Figure 6).

The concomitant Ga 2p_3/2_-peak (Supporting Information File 1, Figure S6) indicates only one Ga species, but we note that the binding energies of the different Ga oxidation states are within 1 eV [50], which does not allow for an unequivocal assignment. The O 1s peaks at 531.30 eV and 531.18 eV clearly show only the presence of organic oxygen and no metal oxides for which the binding energy would have to appear around 529–530 eV [50] (for full XP spectra see Figure S6).

The comparison of the samples after 1 h and 12 h of dispersion shows, that the size of the Ga(Ni) nanoparticles was reduced from 90 ± 20 nm to 30 ± 10 nm, respectively (Figure 4, Figure 5). After the longer dispersion time the fraction of NiGa nanoparticles in the sample with Ga(Ni) particles increases. Evaluating the EDX spectra, the sample after 12 h of dispersion gave a nickel-to-gallium ratio of 38:62 atom % (±1 atom %), while the 1 hour-dispersion time sample was highly Ni deficient with a ratio of 28:72 atom % (±2 atom %). After 24 h of dispersion, the initial 1:1 ratio led to almost exclusively NiGa nanoparticles. Thus, a dispersion time of 24 h before microwave decomposition is needed to gain phase-pure NiGa nanoparticles without Ga(Ni) nanoparticles as by-products. We assume that during the dispersion a chemical reaction of the precursors to Ni/Ga clusters occurs. The formation of clusters from metal precursor materials in ionic liquids has been described before, for example, [EMIm][Ni(P_2_S_8_)] [51], [BMMIm]_16_[Sn_24_Se_56_] [52], [Ru_2_Bi_14_Br_4_][AlCl_4_] [53], [Bi_5_(AlCl_4_)_3_] [53], [Au(Bi_8_)(AlCl_4_)_5_] [54], and [(CuBi_8_)(AlCl_4_)] [55]

In organic solvents, organometallic Ni complexes, e.g., Ni(COD)_2_ and Ni(CO)_4_ are known to form different types of intermetallic clusters with GaCp* and similar compounds, such as Ni(Cp*Ga)(CO)_3_, Ni_4_(Cp*Ga)_4_(CO)_6_, Ni(GaCp*)_4_ [46,56–60]. For example, the reaction of Ni(COD)_2_ with four equivalents of GaCp* in *n*-hexane led to the formation of Ni(GaCp*)_4_ clusters [56]. Thus, the formation of such intermetallic clusters in ionic liquids is a working hypothesis for the required dispersion time of 24 h.

**Figure 7:**
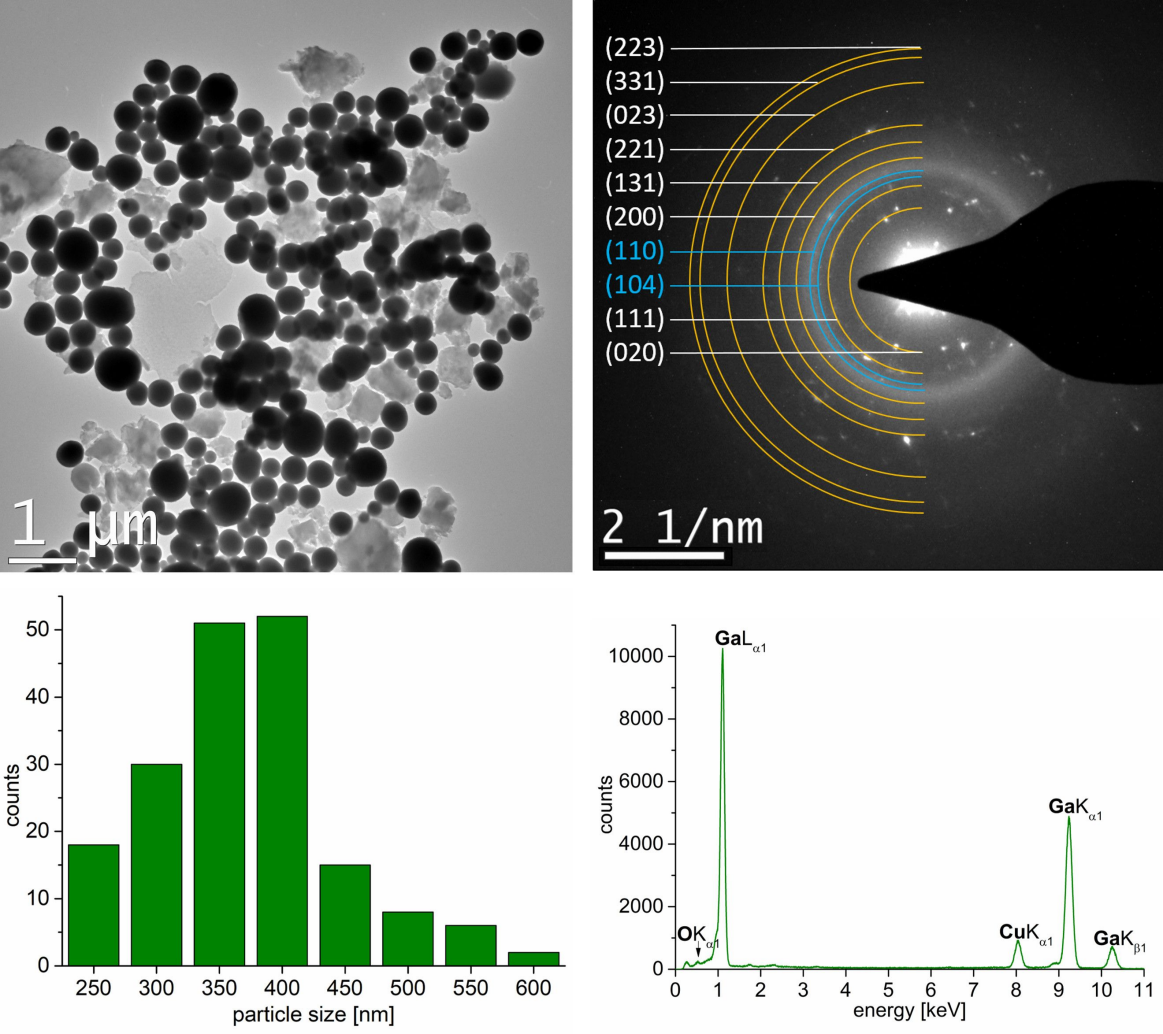
Top: HRTEM images of the Ga(O) nanoparticles from a 0.5 wt % dispersion of GaCp* in [BMIm][NTf_2_] and SAED with indexed reflections for Ga (yellow diffraction rings for space group: *Cmce*) and Ga_2_O_3_ (blue diffraction rings for space group: 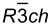). Bottom: Particle size distribution of 350 ± 100 nm (221 particles counted) and EDX spectrum.

To validate the formation of “large” Ga nanoparticles from GaCp*, despite its high decomposition temperature of approximately 300 °C [43], GaCp* was dispersed in [BMIm][NTf_2_] for 24 h prior to thermal decomposition. Through microwave irradiation at 230 °C, a grey powder was obtained after 30 min. The TEM measurements show spherical, crystalline and aggregated nanoparticles with a size distribution of 350 ± 100 nm (Figure 7).

**Figure 8:**
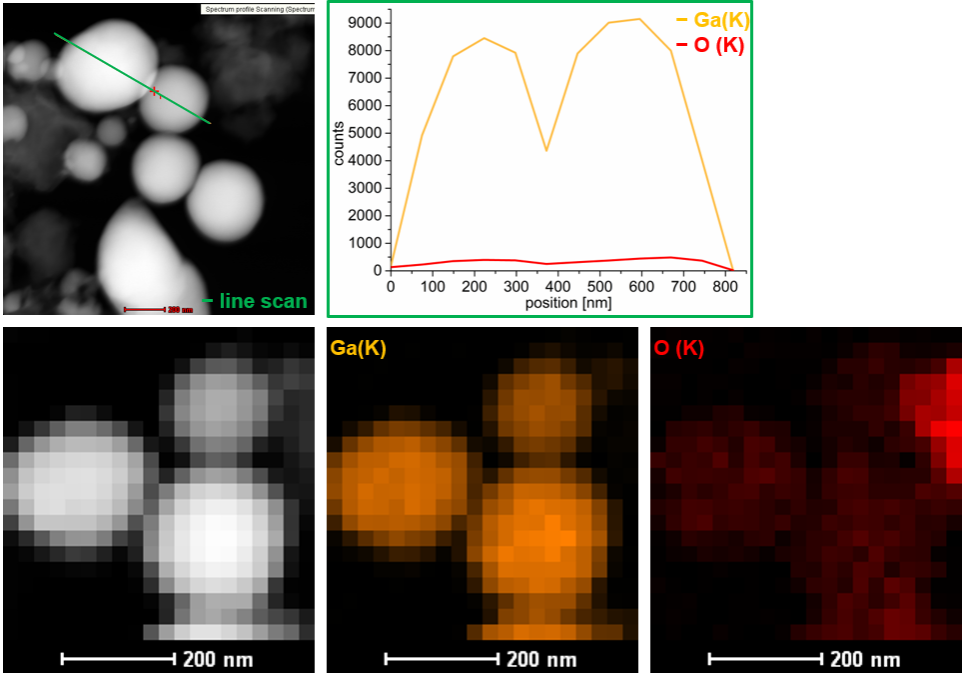
Top: HAADF-STEM images of Ga(O) nanoparticles from 0.5 wt % dispersion of GaCp* in [BMIm][Ntf_2_]. HAADF-STEM-EDX-line-scan (left, green line) with element counts (Ga (K) orange, O (K) red) depending on position (right). Bottom: HAADF-STEM-EDX-mapping: Ga (K) orange, O (K) red.

The SAED-image confirms the formation of the orthorhombic Ga phase (space group: *Cmce*). Two additional reflections can be assigned to the two most intense ones of the Ga_2_O_3_ phase (space group: 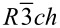). The TEM-EDX indicates a Ga(K)/O(K) ratio of 95:5 ± 4% (Figure 7). This ratio and the subsequent analyses by high-angle annular dark-field (HAADF)-scanning (S)TEM-EDX have to be interpreted very cautiously as EDX is not very well suited for the quantification of elements lighter than fluorine. HAADF-STEM images (Figure 8) were recorded in order to elucidate whether the presence of the oxygen is due to surface oxidation or whether the Ga nanoparticles contain 5% oxygen. An EDX line scan over different particles shows that there is probably no oxide shell around the Ga nanoparticles. Further analysis using EDX mapping also suggests that there is no Ga core–oxide shell structure. Instead, an even distribution of oxygen within the Ga nanoparticles was found (Figure 8).

**Figure 9:**
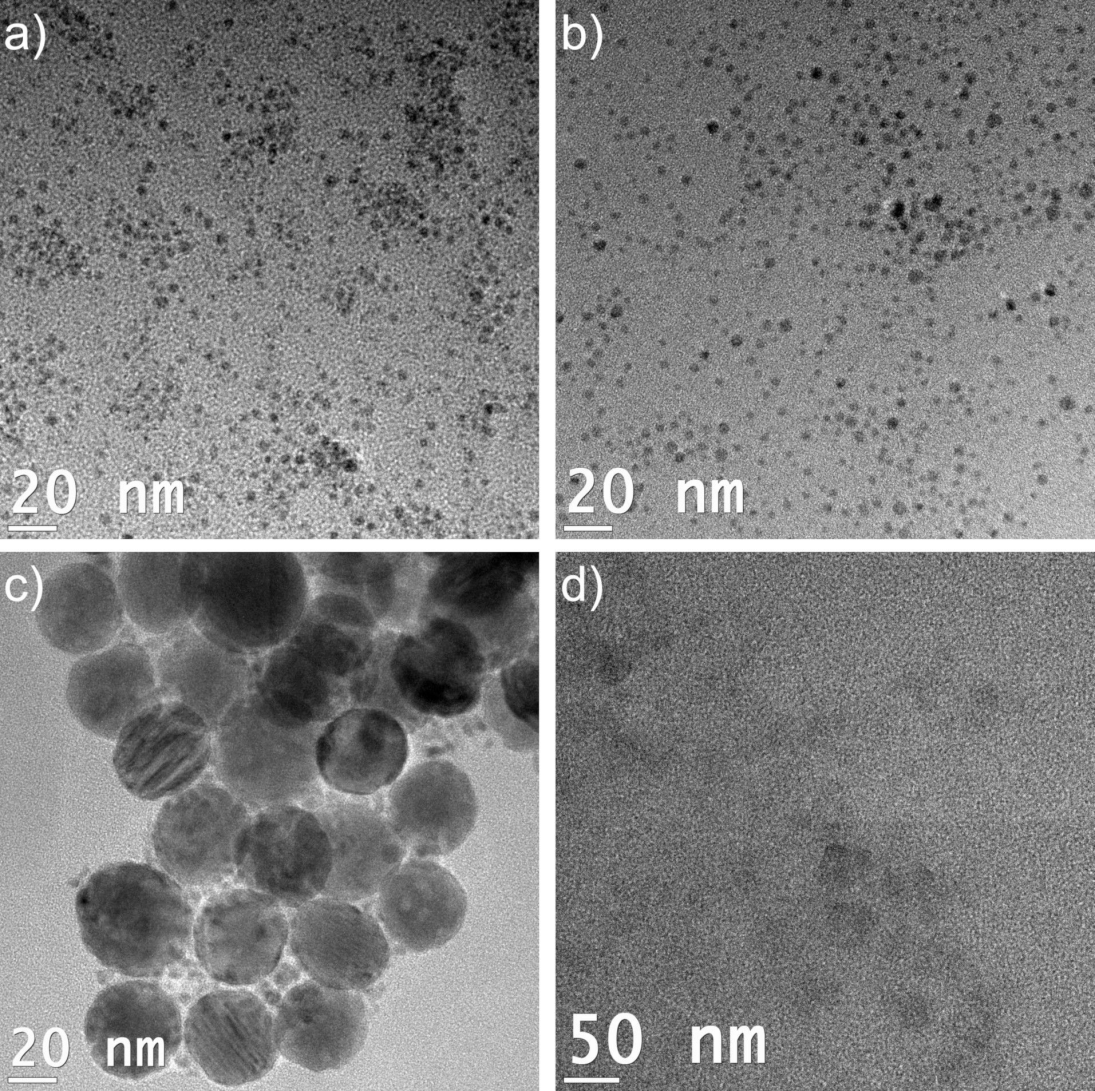
TEM images: a) Ni nanoparticles from 1 wt % dispersion of Ni(COD)_2_ and GaCp* in [EMIm][B(CN)_4_] after 30 min of dispersion and 30 min microwave-induced decomposition. For details of the particle size distribution of 4 ± 1 nm, see Supporting Information File 1, Figure S7. b) Ni/Ga nanoparticles from 1.5 wt % dispersion of Ni(COD)_2_ and GaCp* in [EMIm][B(CN)_4_] after 24 h of dispersion and 30 min of microwave-induced decomposition. For details of the particle size distribution of 4 ± 1 nm, see Supporting Information File 1, Figure S8. c) Ni and Ga nanoparticles from 1 wt % dispersion of Ni(COD)_2_ and GaCp* in [EMIm][BF(CN)_3_] after 1 h of dispersion time and 30 min of microwave-induced decomposition. . For details of the particle size distribution of nickel with 5 ± 1 nm and of gallium with 40 ± 5 nm, see Supporting Information File 1, Figure S9 and Figure S10. d) No nanoparticle formation in [EMIm][FB(CN)_3_] from Ni(COD)_2_ and GaCp* after 24 h of dispersion and 30 min of microwave-induced decomposition.

Furthermore, the cyanoborate ionic liquids [EMIm][B(CN)_4_] and [EMIm][BF(CN)_3_] were tested as a reaction medium for the equimolar ratio of Ni(COD)_2_ and GaCp* with short (0.5 or 1 h) and long (24 h) dispersion times. Following microwave irradiation, TEM images show very different results (Figure 9). After 30 min of dispersion in [EMIm][B(CN)_4_] non-aggregated Ni nanoparticles are formed with a size distribution of 4 ± 1 nm. EDX quantification from three different spots on the TEM grid showed only nickel (see Supporting Information File 1, Figure S7). After 24 h of dispersion in [EMIm][B(CN)_4_] non-aggregated Ni/Ga nanoparticles are formed with a size distribution of 4 ± 1 nm. EDX quantification showed a ratio of nickel to gallium of 38:62 atom % (±2 atom %) (see Supporting Information File 1, Figure S8). In both cases no SAED measurement was possible. In contrast, after 1 h of dispersion in [EMIm][BF(CN)_3_] TEM images showed crystalline particles with two different sizes. The small particles had a size distribution of 5 ± 1 nm and the large particles had a size distribution of 40 ± 5 nm. Through EDX quantification and SAED measurements the small particles were matched to hexagonal Ni (*P*6_3_/*mmc*) and the large particles were matched to cubic Ga (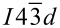) (see Supporting Information File 1, Figure S9 and Figure S10). After 24 h of dispersion in [EMIm][BF(CN)_3_] no nanoparticle formation was observed.

### Catalysis

Previously reported NiGa nanoparticles synthesized in [BMIm][BF_4_] with a size distribution of 14 ± 5 nm were used successfully in the semihydrogenation reaction of the terminal alkyne 1-octyne and the internal alkyne diphenylacetylene, with yields of 90% and selectivities of 94% and 87%, respectively. In this previous work 2 g of the alkyne substrate were mixed with 0.1 g of a 0.5 wt % NiGa@[BMIm][BF_4_] dispersion (containing 3.9 µmol NiGa) in a steel autoclave. Hydrogen was charged with 5 bar at 120 °C and the reaction was run for 3 h [30]. For comparison, the catalysis with NiGa@[BMIm][NTf_2_] was carried out under analogous reaction conditions in the semihydrogenation reaction of the internal alkyne 4-octyne (see below Scheme in Table 2).

**Figure 10:**
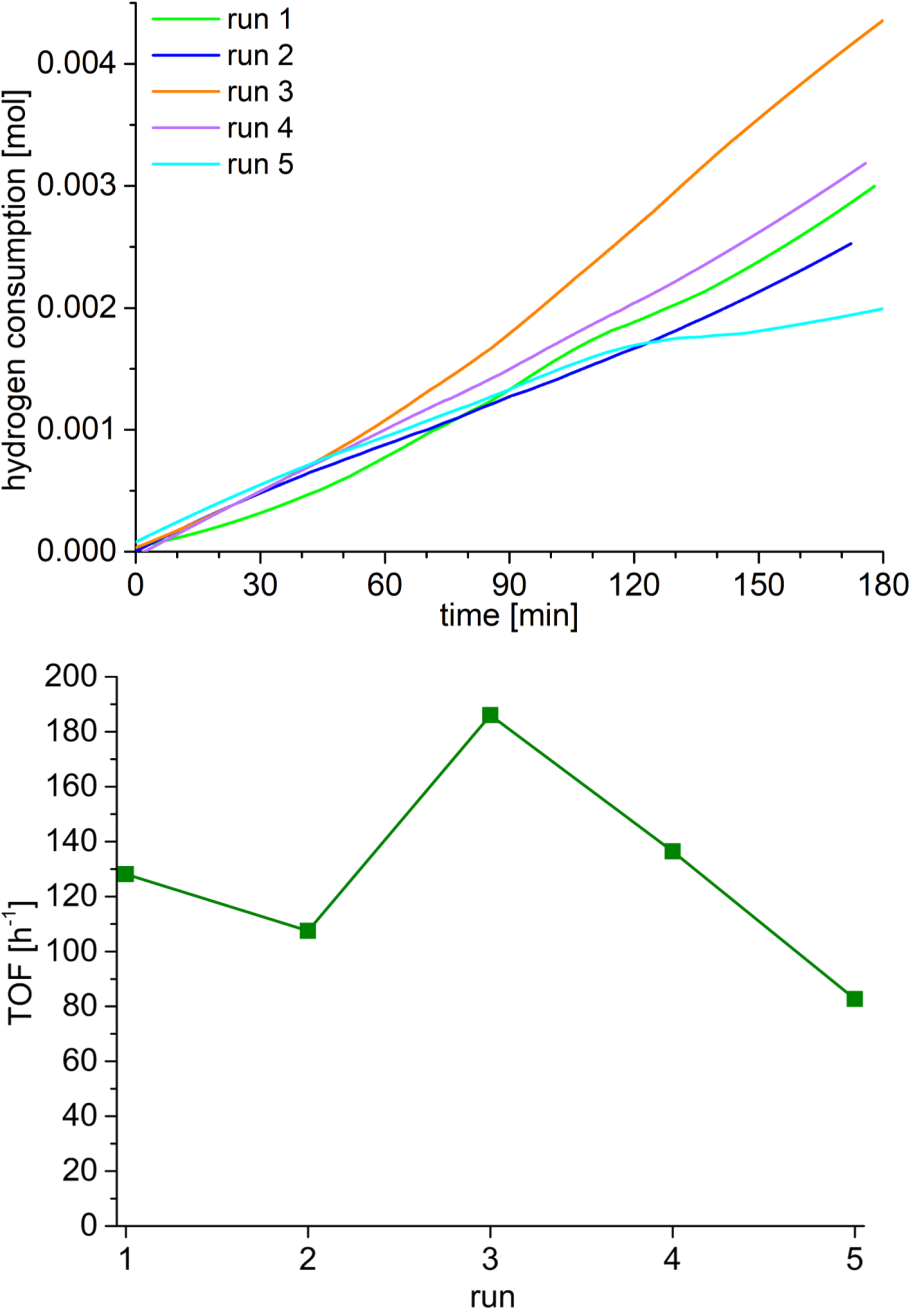
Top: Hydrogen uptake as a function of the time for the semihydrogenation of 0.018 mol 4-octyne (2 g, 2.7 mL) using 0.1 g NiGa@[BMIm][NTf_2_] dispersion (1 wt % = 0.001 g in total metal, 7.8 µmol NiGa) as catalyst at a 4-octyne/metal ratio of 1:2331, 150 °C, 5 bar H_2_, 3 h. A 100% conversion corresponds to an H_2_ uptake of 0.018 mol (36 mg, 403 mL). Bottom: TOF [h^−1^] per run (TOF = mol_substrate_/(mol_catalyst_·time)).

A linear increase of hydrogen consumption is seen in Figure 10. After three hours no plateau value was reached, and the reaction was stopped as the hydrogen consumption was still well below the expected 0.018 mol for a quantitative semihydrogenation. The catalyst was recycled over five runs. In all runs, conversions stayed below 20% (Table 1). TOF values are between 83–186 h^−1^ reaching the highest value at the third run (Figure 10, Table 1).

**Table 1:** Semihydrogenation of 4-octyne to 4-octene using NiGa@[BMIm][NTf_2_].^a^

	run 1a	run 1b^b^	run 2b	run 2b	run 4b	run 5b

conversion	15%	19%	10%	20%	13%	5%
TOF	103 h^−1^	128 h^−1^	107 h^−1^	186 h^−1^	136 h^−1^	83 h^−1^

^a^0.1 g NiGa@[BMIm][NTf_2_] dispersion (1 wt % = 0.001 g in total metal, 7.8 µmol NiGa) and 2 g (2.7 mL, 18.2 mmol) of degassed dry 4-octyne (molar NiGa/substrate ratio = 1:2331) were stirred under 5 bar H_2_ at 120 °C for 3 h. TOF [h^−1^] per run (TOF = mol_substrate_/(mol_catalyst_ · time)). ^b^Runs 1b–5b were carried out with the same catalyst by removing the products in high vacuum.

GC–MS-analysis showed a 100% selectivity towards (*E*)-4-octene in all runs with the NiGa nanoparticles (Table 2).

**Table 2:** Selectivities of the semihydrogenation of 4-octyne to 4-octene using NiGa@[BMIm][NTf_2_].^a^

	run 1a	run 1b^b^	run 2b	run 2b	run 4b	run 5b

(*E*)-4-ene	100%	100%	100%	100%	100%	100%
(*Z*)-4-ene	0%	0%	0%	0%	0%	0%
*n*-octane	0%	0%	0%	0%	0%	0%

Selectivity 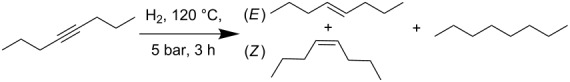						

^a^0.1 g NiGa@[BMIm][NTf_2_] dispersion (1 wt % = 0.001 g in total metal, 7.8 µmol NiGa) and 2 g (2.7 mL, 18.2 mmol) of degassed dry 4-octyne (molar NiGa/substrate ratio = 1:2331) were stirred under 5 bar H_2_ at 120 °C for 3 h. ^b^Runs 1b–5b were carried out with the same catalyst by removing the products in high vacuum.

Utilizing clusters like [Cp*Ru(COD)Cl] in catalytic semihydrogenation reactions, the formation of only (*E*)- [61–63] or (*Z*)- [64–65] derivates are equally known [66]. When metal nanoparticles like the Lindlar catalyst PdPb@CaCO_3_ are used, the formation of (*Z*)*-*alkenes [67–71] is favored. For the formation of (*E*)-alkenes the use of a tandem catalytic system Pd_3_Pb@SiO_2_ + RhSb@SiO_2_ [72] is needed. Catalytic semihydrogenation of internal alkynes favors the formation of (*Z*)-alkenes because of their *syn*-addition style. However, after the initial formation of the *Z*-alkenes, through isomerization reactions the thermodynamically more stable (*E*)-alkenes can be obtained [65,73]. In the literature, the semihydrogenation reaction of the internal alkyne diphenylacetylene with NiGa@[BMIm][BF_4_] led to the formation of a *Z*/*E*-mixture of diphenylethene [30].

During catalysis with nanoparticles in ionic liquids a two-phase system is often formed with the nanoparticles suspended in the denser ionic liquid in the lower phase and the organic substrate in the upper phase. Studies have shown that catalyses in ionic liquids are slower due to diffusion limitations and, thus, lower conversion rates are obtained than in solventless systems [61]. Still, catalysis in ionic liquids achieves the same selectivities. Also, the IL prevents nanoparticle agglomeration to allow for catalyst recycling over several runs [61]. To examine the influence of the ionic liquid, the reaction was repeated under solventless conditions with precipitated, largely IL-free NiGa nanoparticles. The NiGa nanoparticles were precipitated from the IL with acetonitrile and the IL was removed as much as possible by washing with acetonitrile (see Supporting Information File 1, Figure S11). The precipitated NiGa nanoparticles exhibited over three runs very high conversion rates of 82–96% (Table 3).

**Table 3:** Semihydrogenation of 4-octyne to 4-octene using precipitated IL-free NiGa nanoparticles.^a^

	run 1	run 2	run 3

conversion^b^	93–96%	92–93%	82–92%
TOF	170 h^−1^	211 h^−1^	154 h^−1^

^a^10 mg precipitated, IL-free NiGa nanoparticles (77 µmol) and 1 g (1.35 mL, 9 mmol) of degassed dry 4-octyne (molar NiGa/substrate ratio = 1:115) were stirred under 5 bar H_2_ at 120 °C. Runs 1–3 were carried out with the same catalyst by removing the products in high vacuum. TOF [h^−1^] per run (TOF = mol_substrate_/(mol_catalyst_ · time)). ^b^Runs 1–3 were carried out twice with the same catalyst by removing the products in high vacuum. For each of the two runs the conversion values are given.

**Figure 11:**
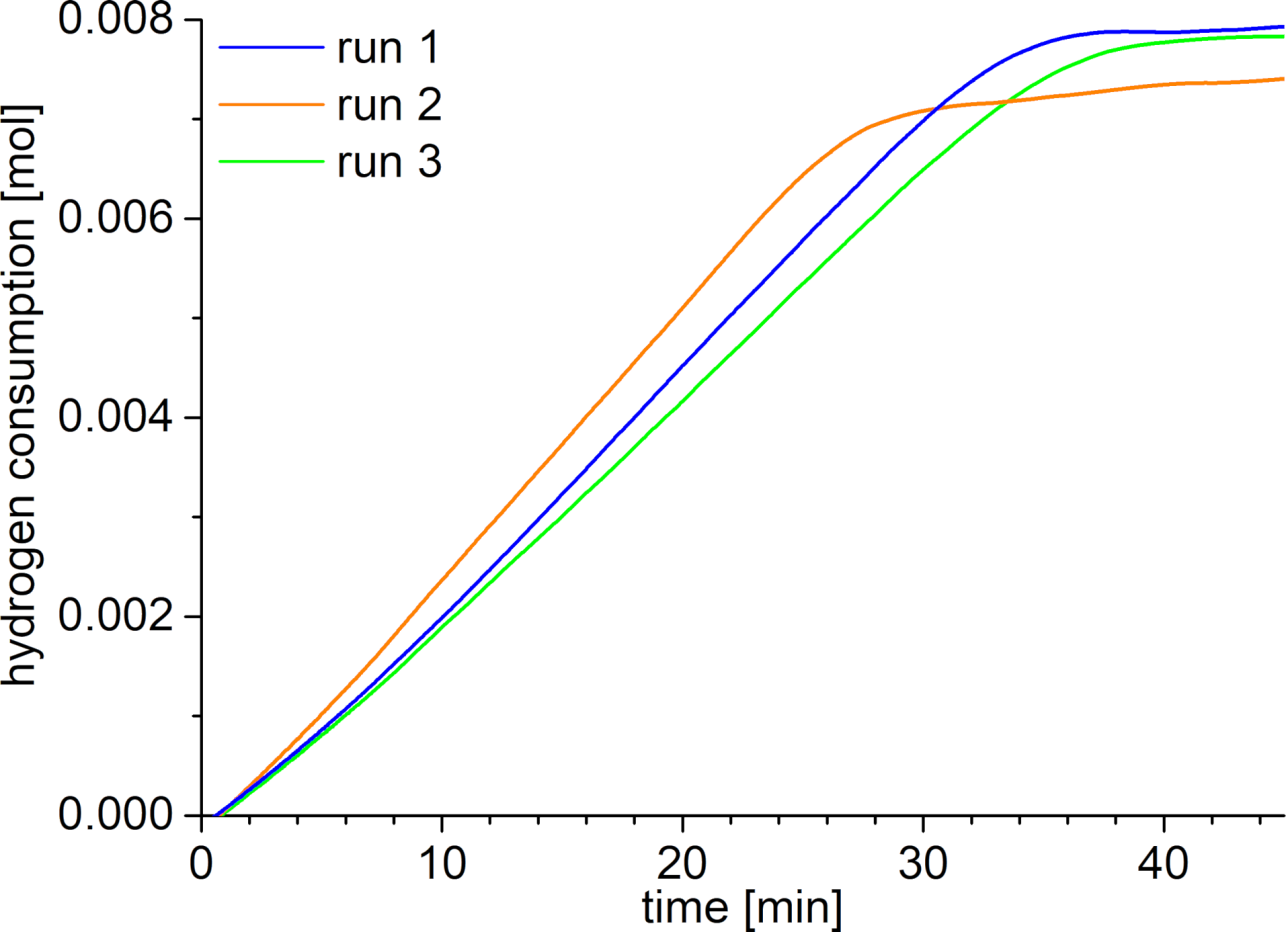
Hydrogen uptake as a function of the time for the semihydrogenation of 0.009 mol 4-octyne (1 g, 1.35 mL) using 10 mg precipitated, IL-free precipitated NiGa nanoparticles (77 µmol NiGa) as catalyst at a 4-octyne/metal ratio of 1:115, 150 °C, 5 bar H_2_. A 100% conversion corresponds to an H_2_ uptake of 0.009 mol (18 mg, 202 mL).

After 30 min the hydrogen consumption reaches a plateau at an H_2_ uptake value that typically corresponds to over 90% conversion (Figure 11, Table 3).

The selectivity towards the alkene still remains near 90% and can approach 100% (Table 4). Taking a closer look at the GC–MS results, *E*/*Z*-selectivity of 4-octene can be distinguished, as well as a bond-shift isomerization reaction to *E*/*Z*-3-octene. Bond-shift isomerization reactions towards 3-octene are dominant in the first run. In this first run also a 1:0.73 mol/mol *E*/*Z*-mixture of 4-octene was formed. In the second and third run, *E*-4-octene was the predominant product.

**Table 4:** Selectivities of the semihydrogenation of 4-octyne to 4-octene using precipitated, IL-free NiGa nanoparticles.^a^

	run 1	run 2	run 3

(*E*)-4-ene^b^	46–48%	87–92%	82–88%
(*Z*)-4-ene^b^	32–34%	0%	0%
(*E*)-3-ene^b^	4–6%	0%	0%
(*Z*)-3-ene^b^	7–12%	0–5%	0–4%
*n*-octane	0%	0%	0%

Selectivity 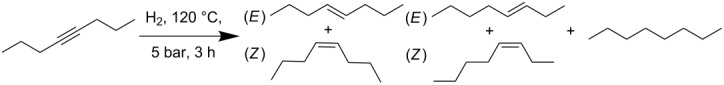			

^a^10 mg precipitated, IL-free NiGa nanoparticles (77 µmol) and 1 g (1.35 mL, 9 mmol) of degassed dry 4-octyne (molar NiGa/substrate ratio = 1:115) were stirred under 5 bar H_2_ at 120 °C. Runs 1–3 were carried out with the same catalyst by removing the products in high vacuum. ^b^Runs 1–3 were carried out twice with the same catalyst by removing the products in high vacuum. For each of the two runs the composition values are given.

In comparison, precipitated NiGa nanoparticles have higher TOF values as NiGa @[BMIm][NTf_2_] (compare Table 1 and Table 3, Supporting Information File 1, Table S3). TOF values are slightly increased for precipitated, IL-free NiGa nanoparticles.

**Figure 12:**
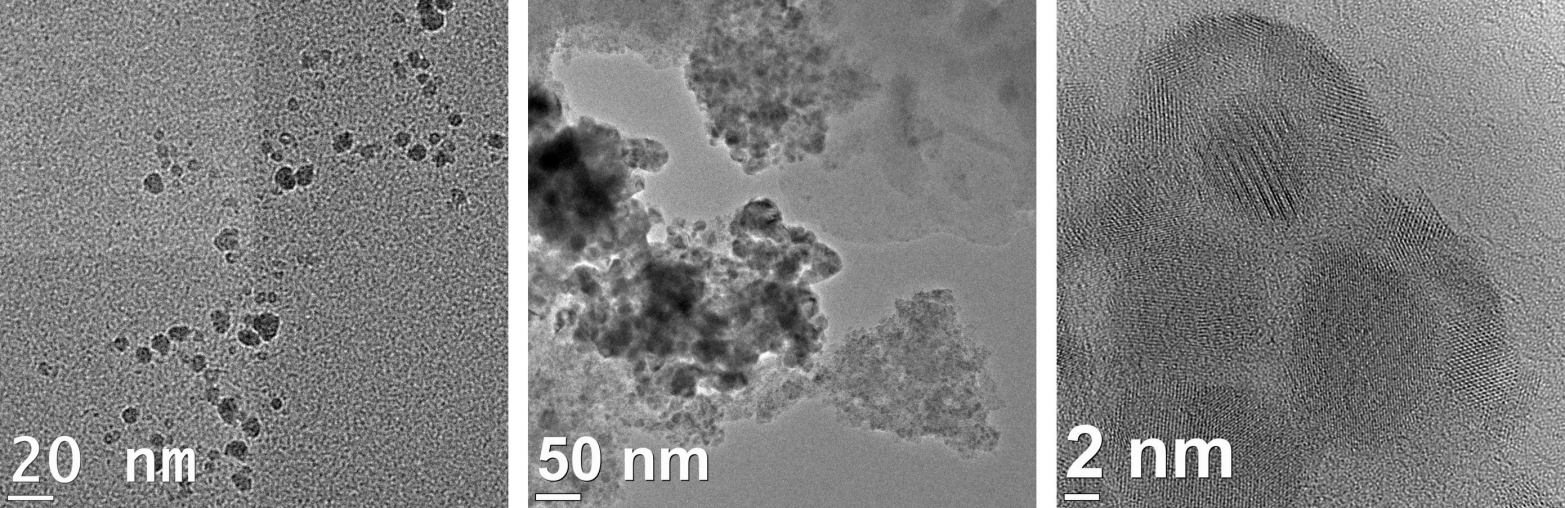
HRTEM image of precipitated NiGa nanoparticles before the catalysis (left) and highly agglomerated NiGa nanoparticles after the catalysis (middle and right).

To determine whether the precipitated NiGa nanoparticles used in the catalytic reaction change over time HRTEM images are measured (Figure 12). After three runs, the particles are more agglomerated, but their size distribution did not change. Particles are still at a size of 5 ± 1 nm.

## Conclusion

After 24 h of dispersion of all-hydrocarbon precursors Ni(COD)_2_ and GaCp* in the ionic liquid [BMIm][NTf_2_], microwave-induced co-decomposition yielded phase-pure NiGa nanoparticles of 5 ± 1 nm. In order to gain crystalline NiGa nanoparticles, 30 min of microwave-induced decomposition were found to be required. With dispersion times of 1 or 12 h before the microwave-induced decomposition, Ga(Ni) nanoparticles were formed as a by-product to NiGa nanoparticles. To complete this investigation, GaCp* was successfully decomposed in [BMIm][NTf_2_] to Ga_2_O_3_-doped Ga particles with a size of 350 ± 100 nm. The formation of core–shell sparticles can be ruled out by HRTEM/STEM-EDX-measurements.

Phase-pure NiGa nanoparticles were tested in the semihydrogenation of an internal alkyne. A comparison study between NiGa nanoparticles in ionic liquid and precipitated NiGa nanoparticles under solventless conditions was performed. NiGa@[BMIm][NTf_2_] catalyzed the hydrogenation of the internal alkyne 4-octyne with 100 % selectivity towards *E*-4-octene over 5 runs, but with poor conversions. After the removal of the IL, precipitated NiGa nanoparticles achieved an increased conversion higher than 90% over 3 runs. The selectivities towards the alkene still reached 100%.

## Experimental

Due to the sensitivity of the precursor substances towards hydrolysis and oxidation, that is, moisture and oxygen (air), all experiments were carried out in a purified argon or nitrogen atmosphere by using standard Schlenk techniques. Samples were prepared and stored in an MBraun Glovebox. Solvents (acetonitrile, *n*-hexane, and methylene chloride) were dried by using an MBraun solvent purification system or 4 Å molecular sieves (1-methylimidazole, 1-chlorobutane) and stored over 4 Å molecular sieves in a nitrogen atmosphere. Remaining water contents of the solvents were measured by a coulometric Karl-Fischer titration (ECH/ANALYTIK JENA AQUA 40.00) and did not exceed 10 ppm.

Ni(COD)_2_ was purchased from ABCR, stored at −4 °C and used without further purification. GaCp* was synthesized according to literature under strictly inert dry argon conditions [69]. The ionic liquid [BMIm][NTf_2_] was synthesized according to the literature by reacting 1-methylimidazole with 1-chlorobutane to yield first [BMIm][Cl], which was further reacted with LiNTf_2_ to give [BMIm][NTf_2_] [74–75]. The IL was dried under ultra-high vacuum (10^−7^ mbar) at 80 °C for three days. [EMIm][B(CN)_4_] and [EMIm][BF(CN)_3_] was synthesized similarly by metathesis reaction of [EMIm][Br] with K[B(CN)_4_] and K[BF(CN)_3_], respectively [76–77]. Propylene carbonate was dried over 4 Å molecular sieves for several days. Characterization was carried out by ^1^H and ^13^C NMR spectroscopy. Quantitative anion exchange and IL purity of 99.9% was assessed by ion chromatography (Dionex ICS-1100, with IonPac^®^ AS22, 4 × 250 mm column). The water content measured by coulometric Karl-Fischer titration was below 10 ppm.

**Powder X-ray diffraction.** PXRD data were obtained at ambient temperature on a Bruker D2 Phaser using a flat sample holder and Cu Kα radiation (λ = 1.54182 Å, 35 kV). Samples had been precipitated with acetonitrile from the nanoparticle/IL dispersion and washed several times with acetonitrile. PXRDs were measured for 1 h.

**Transmission electron microscopy.** TEM was performed with a FEI Tecnai G2 F20 electron microscope [78] operated at 200 kV accelerating voltage, with a FEI Titan 80-300 TEM operated at 300 kV accelerating voltage [79] or with a Philips CM20 operated at 200 kV accelerating voltage. Conventional TEM images were recorded with a Gatan UltraScan 1000P detector.

**Energy-dispersive X-ray spectroscopy.** EDX spectra for elemental (metal) analysis were recorded using an exposure time of 3 min. High-angle annular dark-field scanning transmission electron microscopy (HAADF-STEM) was also performed with the FEI Tecnai G2 F20 electron microscope. All EDX spectra acquired in STEM mode were averaged scans over selected areas of about 100 × 100 nm^2^. The EDX spectra of an isolated particle were measured at several points with a spatial resolution of 1 nm^2^ (acquisition time of 30 s at each point). The instrumental errors of this high-resolution EDX scan led to an estimated standard deviation of 10–15% relative error. TEM samples were prepared by drop-casting the diluted material on 200 µm carbon-coated copper or gold grids. The size distribution was determined manually or with the aid of the Gatan Digital Micrograph software from at least 50 individual particles.

**Selected-area electron diffraction (SAED)** patterns have been recorded with the above mentioned TEM instruments. The area selection was achieved with a round aperture moved in the first intermediate image plane with a corresponding diameter of 0.64 µm in the object plane. For each acquisition a sample region with a significant amount of material was placed inside the aperture. The objected was illuminated with a wide-spread parallel beam obtaining focused diffraction patterns. The diffraction images were calibrated with Debye–Scherrer patterns recorded from a gold reference sample (S106, Plano GmbH, Wetzlar, Germany).

**High-resolution X-ray photoelectron spectroscopy, HRXPS- (ESCA-)** measurements were performed with a Fisons/VG Scientific ESCALAB 200X XP-spectrometer, operating at 70–80 °C, a pressure of 7.0 × 10^–9^ bar and a sample angle of 33°. Spectra were recorded using polychromatic Al Kα excitation (11 kV, 20 mA) and an emission angle of 0°. Calibration of the XPS was carried out by recording spectra with Al Kα X-rays from clean samples of copper, silver and gold at 50 eV and 10 eV pass energy and comparison with reference values. Spectra were obtained with an Al Kα X-ray source, using C 1s as a reference for the binding energy [50].

**Gas chromatography–mass spectrometry (GC–MS)** data were recorded on a Thermo Finnigan Trace DSQ (Shimadzu GC2014, column Ultra2, crosslinked 5% PhMe silicone, 25 m × 0.2 mm × 11 mm).

### Preparation of nanoparticles in ionic liquid

Syntheses of Ni/Ga nanoparticles were prepared in septum-sealed 10 mL CEM microwave-vials in a CEM Discover microwave under argon atmosphere. Ni(COD)_2_ and GaCp* were suspended for a chosen time (30 min, 1 h, 12 h or 24 h) in the dried and desoxygenated IL or PC before microwave decomposition to gain 0.5–1.5 wt % dispersion of the nanoparticles. All precursor dispersions were decomposed at a power of 50 W to a temperature of 230 °C for a chosen time (10 min, 20 min, 30 min). For specific mass values, dispersion and decomposition times see Supporting Information File 1, Table S4. In the case of Ga(O) nanoparticles in [BMIm][NTf_2_] GaCp* (15.6 mg, 0.076 mmol) was suspended for 24 h in the dried and desoxygenated [BMIm][NTf_2_] (1 g, 0.71 mL, density = 1.41 g/cm^3^) before microwave decomposition (60 min, 50 W, 230 °C) to gain 0.5 wt % nanoparticles in ionic liquid.

### Catalytic hydrogenation of alkynes

A Büchi stainless steel autoclave with a glass inlet was charged with 0.1 g of freshly synthesized NiGa@[BMIm][NTf_2_] dispersion (1 wt % in total metal, 8 µmol NiGa). 2 g of degassed, dry substrate 4-octyne (2.7 mL, 18.2 mmol) was added. For the hydrogenation without ionic liquid 10 mg precipitated NiGa nanoparticles (0.86 mmol) were mixed with 1 g of degassed, dry substrate 4-octyne (1.3 mL, 9.1 mmol). The reaction mixture was heated to 120 °C. After reaching the reaction temperature, the autoclave was pressurized with 5 bar H_2_ (Büchi press flow gas controller, bpc), which was kept constant by the Büchi bpc. After reaching a plateau value or after a maximum time of 3 h the reaction was stopped, the autoclave was cooled down and a 0.5 g sample was analyzed for its content by GC/MS and NMR. Conversion and selectivity were determined by GC/MS [retention times in min: 1.67 (octane), 1.75 ((*Z*)-4-octene), 1.78 ((*E*)-4-octene), 1.86 ((*Z*)-3-octene), 1.94 ((*E*)-3-octene), 2.29 (4-octyne), Shimadzu GC2014, column Ultra2, crosslinked 5% PhMe silicone, 25 m × 0.2 mm × 11 mm].

## Supporting Information

The supporting information contains further analysis of Ni/Ga nanoparticles in the ionic liquids [BMIm][BF_4_], [EMIm][B(CN)_4_], and [EMIm][BF(CN)_3_], and in propylene carbonate after different dispersion times prior to the decomposition and different time periods of microwave-induced decomposition. Furthermore, additional particle size distributions, XP spectra and catalytic results, as well as particle preparation descriptions are given.

File 1 Additional experimental details.

## References

[1] Wu S-H, Chen D-H (2003). J Colloid Interface Sci.

[2] Luo X, Chen Y, Yue G-H, Peng D-L, Luo X (2009). J Alloys Compd.

[3] Golindano T d C, Martínez S I, Delgado O Z, Rivas G P (2005). NSTI Nanotech.

[4] Alonso F, Riente P, Yus M (2009). Eur J Org Chem.

[5] Park J, Kang E, Son S U, Park H M, Lee M K, Kim J, Kim K W, Noh H-J, Park J-H, Bae C J (2005). Adv Mater (Weinheim, Ger).

[6] Reina A, Favier I, Pradel C, Gómez M (2018). Adv Synth Catal.

[7] Alonso F, Riente P, Yus M (2009). Tetrahedron.

[8] Dhakshinamoorthy A, Pitchumani K (2008). Tetrahedron Lett.

[9] Jiang H-y, Zhang S-s, Sun B (2018). Catal Lett.

[10] Wang L, Li F, Chen Y, Chen J (2019). J Energy Chem.

[11] Trimm D L, Liu I O Y, Cant N W (2010). Appl Catal, A.

[12] Wang H, Li X, Li M, Xie K, Liao L (2015). Beilstein J Nanotechnol.

[13] Tzitzios V, Basina G, Gjoka M, Alexandrakis V, Georgakilas V, Niarchos D, Boukos N, Petridis D (2006). Nanotechnology.

[14] Vorobjova A I, Shimanovich D L, Yanushkevich K I, Prischepa S L, Outkina E A (2016). Beilstein J Nanotechnol.

[15] Gong J, Wang L L, Liu Y, Yang J H, Zong Z G (2008). J Alloys Compd.

[16] Fernández G, Sort J, Pleixats R (2018). ChemistrySelect.

[17] Schunn R A, Ittel S D, Cushing M A, Baker R, Gilbert R J, Madden D P (2007). Inorg Synth.

[18] Vollmer C, Redel E, Abu-Shandi K, Thomann R, Manyar H, Hardacre C, Janiak C (2010). Chem – Eur J.

[19] Siebels M, Mai L, Schmolke L, Schütte K, Barthel J, Yue J, Thomas J, Smarsly B M, Devi A, Fischer R A (2018). Beilstein J Nanotechnol.

[20] Konnerth H, Prechtl M H G (2017). New J Chem.

[21] Wegner S, Rutz C, Schütte K, Barthel J, Bushmelev A, Schmidt A, Dilchert K, Fischer R A, Janiak C (2017). Chem – Eur J.

[22] Migowski P, Machado G, Texeira S R, Alves M C M, Morais J, Traverse A, Dupont J (2007). Phys Chem Chem Phys.

[23] Borodziński A, Bond G C (2008). Catal Rev: Sci Eng.

[24] Maurer B R, Galobardes M (1989). Selective hydrogenation of phenylacetylene in the presence of styrene. U.S. Patent.

[25] Molnár Á, Sárkány A, Varga M (2001). J Mol Catal A: Chem.

[26] Armbrüster M, Kovnir K, Behrens M, Teschner D, Grin Y, Schlögl R (2010). J Am Chem Soc.

[27] Armbrüster M, Wowsnick G, Friedrich M, Heggen M, Cardoso-Gil R (2011). J Am Chem Soc.

[28] Krajčı M, Hafner J (2012). J Catal.

[29] Desai S P, Ye J, Zheng J, Ferrandon M S, Webber T E, Platero-Prats A E, Duan J, Garcia-Holley P, Camaioni D M, Chapman K W (2018). J Am Chem Soc.

[30] Schütte K, Doddi A, Kroll C, Meyer H, Wiktor C, Gemel C, van Tendeloo G, Fischer R A, Janiak C (2014). Nanoscale.

[31] Li C, Chen Y, Zhang S, Zhou J, Wang F, He S, Wei M, Evans D G, Duan X (2014). ChemCatChem.

[32] Hu M, Yang W, Liu S, Zhu W, Li Y, Hu B, Chen Z, Shen R, Cheong W-C, Wang Y (2019). Chem Sci.

[33] Feschotte P, Eggimann P (1979). J Less-Common Met.

[34] Micke K, Markovski S L, Ipser H, van Loo F J J (1998). Ber Bunsen-Ges.

[35] Okamoto H (2010). J Phase Equilib Diffus.

[36] Yuan W X, Qiao Z Y, Ipser H, Eriksson G (2004). J Phase Equilib Diffus.

[37] Studt F, Sharafutdinov I, Abild-Pedersen F, Elkjær C F, Hummelshøj J S, Dahl S, Chorkendorff I, Nørskov J K (2014). Nat Chem.

[38] Sharafutdinov I, Elkjær C F, Pereira de Carvalho H W, Gardini D, Chiarello G L, Damsgaard C D, Wagner J B, Grunwaldt J-D, Dahl S, Chorkendorff I (2014). J Catal.

[39] Torelli D A, Francis S A, Crompton J C, Javier A, Thompson J R, Brunschwig B S, Soriaga M P, Lewis N S (2016). ACS Catal.

[40] Chiang C L, Lin K S, Lin Y G (2017). Top Catal.

[41] Tang Q, Shen Z, Huang L, He T, Adidharma H, Russell A G, Fan M (2017). Phys Chem Chem Phys.

[42] Liu Y, Liu X, Feng Q, He D, Zhang L, Lian C, Shen R, Zhao G, Ji Y, Wang D (2016). Adv Mater (Weinheim, Ger).

[43] Cokoja M, Parala H, Schröter M-K, Birkner A, van den Berg M W E, Grünert W, Fischer R A (2006). Chem Mater.

[44] Scholten J D, Ebeling G, Dupont J (2007). Dalton Trans.

[45] Clement N D, Cavell K J, Jones C, Elsevier C J (2004). Angew Chem.

[46] Cadenbach T, Gemel C, Schmid R, Halbherr M, Yusenko K, Cokoja M, Fischer R A (2009). Angew Chem.

[47] Gutel T, Garcia-Antõn J, Pelzer K, Philippot K, Santini C C, Chauvin Y, Chaudret B, Basset J-M (2007). J Mater Chem.

[48] Bilecka I, Niederberger M (2010). Nanoscale.

[49] Kölle U, Khouzami F, Lueken H (1982). Chem Ber.

[50] Moulder J F, Stickle W F, Sobol P (1992). Handbook of X–ray Photoelectron Spectroscopy.

[51] Santner S, Heine J, Dehnen S (2016). Angew Chem.

[52] Thiele G, Santner S, Dehnen S (2017). Z Kristallogr - Cryst Mater.

[53] Groh M F, Isaeva A, Ruck M (2012). Chem – Eur J.

[54] Müller U, Isaeva A, Richter J, Knies M, Ruck M (2016). Eur J Inorg Chem.

[55] Knies M, Kaiser M, Isaeva A, Müller U, Doert T, Ruck M (2018). Chem – Eur J.

[56] Jutzi P, Neumann B, Reumann G, Stammler H-G (1998). Organometallics.

[57] Jutzi P, Neumann B, Reumann G, Schebaum L O, Stammler H-G (1999). Organometallics.

[58] Buchin B, Steinke T, Gemel C, Cadenbach T, Fischer R A (2005). Z Anorg Allg Chem.

[59] Gemel C, Steinke T, Cokoja M, Kempter A, Fischer R (2004). Eur J Inorg Chem.

[60] Steinke T, Gemel C, Cokoja M, Winter M, Fischer R A (2004). Angew Chem.

[61] Fürstner A (2019). J Am Chem Soc.

[62] Karunananda M K, Mankad N P (2015). J Am Chem Soc.

[63] Zhou Y-P, Mo Z, Luecke M-P, Driess M (2018). Chem – Eur J.

[64] Becica J, Glaze O D, Wozniak D I, Dobereiner G E (2018). Organometallics.

[65] Hauwert P, Maestri G, Sprengers J W, Catellani M, Elsevier C J (2008). Angew Chem.

[66] Kusy R, Grela K (2016). Org Lett.

[67] Kluwer A M, Koblenz T S, Jonischkeit T, Woelk K, Elsevier C J (2005). J Am Chem Soc.

[68] Lee J-K, Kim D-W, Cheong M-S, Lee H-J, Cho B-W, Kim H-S, Mukherjee D (2010). Bull Korean Chem Soc.

[69] Savoia D, Tagliavini E, Trombini C, Umani-Ronchi A (1981). J Org Chem.

[70] Schwab F, Weidler N, Lucas M, Claus P (2014). Chem Commun.

[71] Wagh Y S, Asao N (2015). J Org Chem.

[72] Furukawa S, Komatsu T (2016). ACS Catal.

[73] Tokmic K, Fout A R (2016). J Am Chem Soc.

[74] Bonhôte P, Dias A-P, Papageorgiou N, Kalyanasundaram K, Grätzel M (1996). Inorg Chem.

[75] Burrell A K, Sesto R E D, Baker S N, McCleskey T M, Baker G A (2007). Green Chem.

[76] Ignat’ev N V, Finze M, Sprenger J A P, Kerpen C, Bernhardt E, Willner H (2015). J Fluorine Chem.

[77] Ignat'ev N V, Finze M (2019). Eur J Inorg Chem.

[78] Luysberg M, Heggen M, Tillmann K (2016). J Large-Scale Res Facil.

[79] Thust A, Barthel J, Tillmann K (2016). J Large-Scale Res Facil.

