# Supplementary material for: Synthesis of nickel/gallium nanoalloys using a dual-source approach in 1-alkyl-3-methylimidazole ionic liquids
Source: Beilstein J Nanotechnol. 2019 Aug 21;10:1754–67. doi: 10.3762/bjnano.10.171 (PMC6720474; doi:10.3762/bjnano.10.171)
Supplement: File 1 — Additional experimental details. [file Beilstein_J_Nanotechnol-10-1754-s001.pdf]

BEILSTEIN JOURNAL OF NANOTECHNOLOGY

Supporting Information

for

Synthesis of nickel/gallium nanoalloys using a dual-source approach in 1-alkyl-3-methylimidazole ionic liquids

Ilka Simon, Julius Hornung, Juri Barthel, Jörg Thomas, Maik Finze, Roland A. Fischer and Christoph Janiak

Beilstein J. Nanotechnol. **2019,** *10,* 1754–1767. doi:10.3762/bjnano.10.171

Additional experimental details

License and Terms: This is a supporting information file under the terms of the Creative Commons Attribution License (http://creativecommons.org/licenses/by/4.0). Please note that the reuse, redistribution and reproduction in particular requires that the authors and source are credited.
The license is subject to the *Beilstein Journal of Nanotechnology* terms and conditions: (https://www.beilstein-journals.org/bjnano)

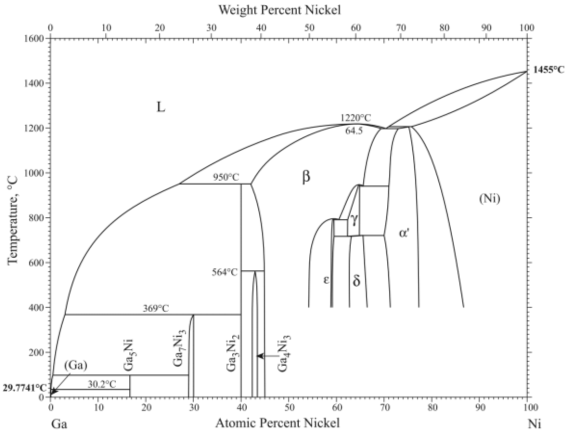

Phase	Composition, at.% Ni	Pearson symbol	Space group	Strukturbericht designation	Prototype
(Ga)	0	*oC*8	*Cmca*	*A*11	Ga
Ga_5Ni	16.7	…	…	…	…
Ga_7Ni_3	29-30	*cI*40	$Im\bar{3}m$	$D8_f$	Ge_7Ir_3
Ga_3Ni_2	40	*hP*5	$P\bar{3}m1$	$D5_{13}$	Al_3Ni_2
Ga_4Ni_3	42.4-43.4	*cI*112	*Ia*3*d*	…	…
β	42-69.4	*cP*2	$Pm\bar{3}m$	*B*2	CsCl
ε	59-59.5	…	…	…	…
γ	62.5-65	*hP*4	$P6_3/mmc$	$B8_1$	NiAs
δ	63-66.5	*oC*16	*Cmmm*	…	Ga_3Pt_5
α′	70-77	*cP*4	$Pm\bar{3}m$	$L1_2$	$AuCu_3$
(Ni)	75.7-100	*cF*4	$Fm\bar{3}m$	*A*1	Cu

Figure S1: Phase diagram and crystal structure data for Ni/Ga taken from literature [1].

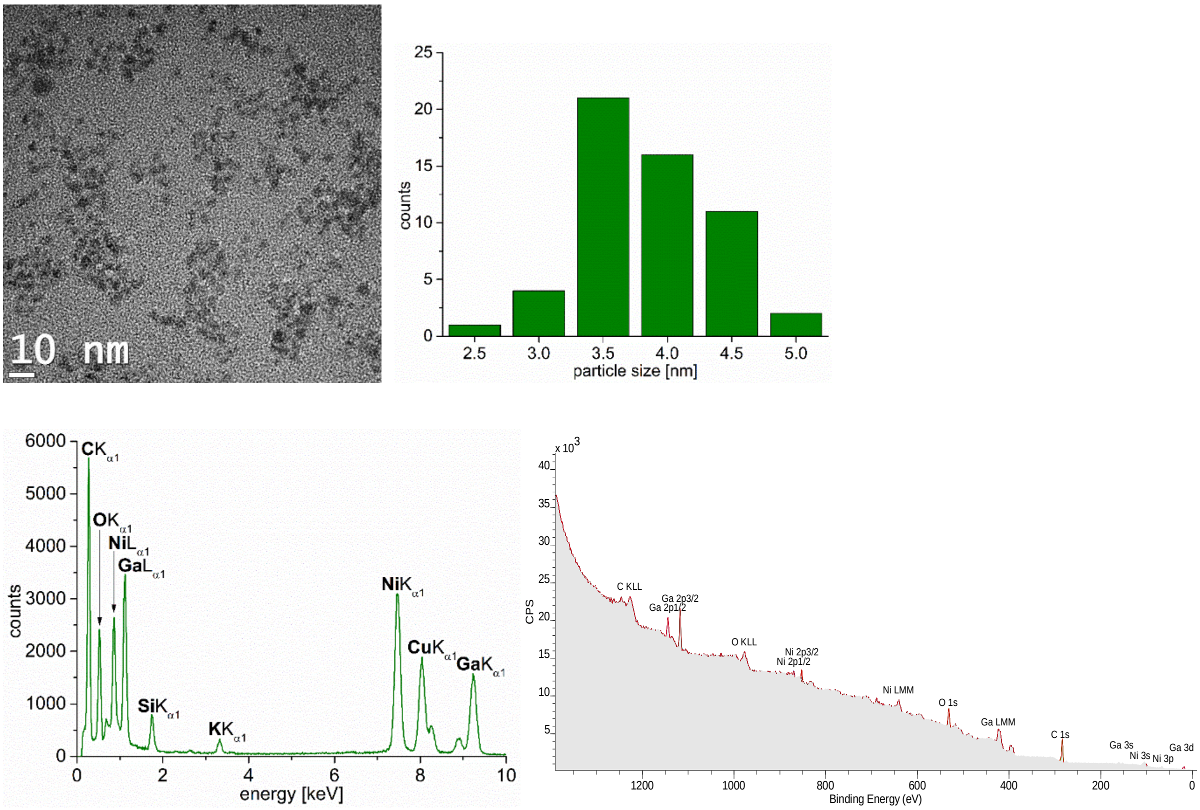

Figure S2: HR-TEM-Images and particle size distribution of the Ni/Ga-nanoparticles from 1 wt % dispersion of $Ni(COD)_2$ and GaCp* in [BMIm][BF_4] (top) with 24 hours dispersion time, 10 minutes microwave decomposition time. Particle size 2.5 ± 0.5 nm (65 particles counted). EDX- and XP-spectra (bottom). EDX-Quantification from three different spots on the TEM-Grid showed equimolar ratio of nickel to gallium of 41:59 atom % (±1 atom %). No SAED-measurement was possible.

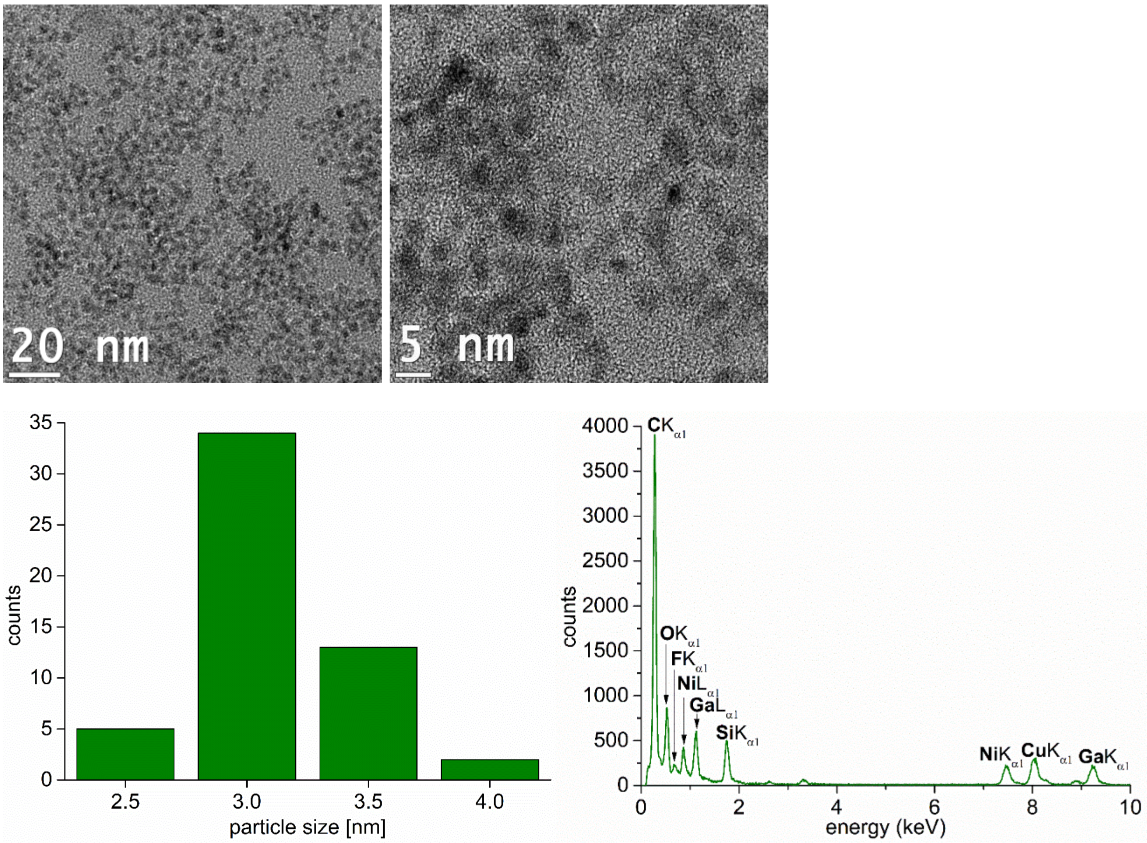

Figure S3: HR-TEM-Images of the Ni/Ga-nanoparticles from 0.5 wt % dispersion of $Ni(COD)_2$ and GaCp* in $[BMIm][BF_4]$ (top) with 24 hours dispersion time, 20 minutes microwave decomposition. Particle size distribution 2.5 ± 0.5 nm (56 particles counted) and EDX-spectra (bottom). EDX-Quantification from three different spots on the TEM-Grid showed equimolar ratio of nickel to gallium of 57:43 atom % (±2 atom %). No SAED-measurement was possible.

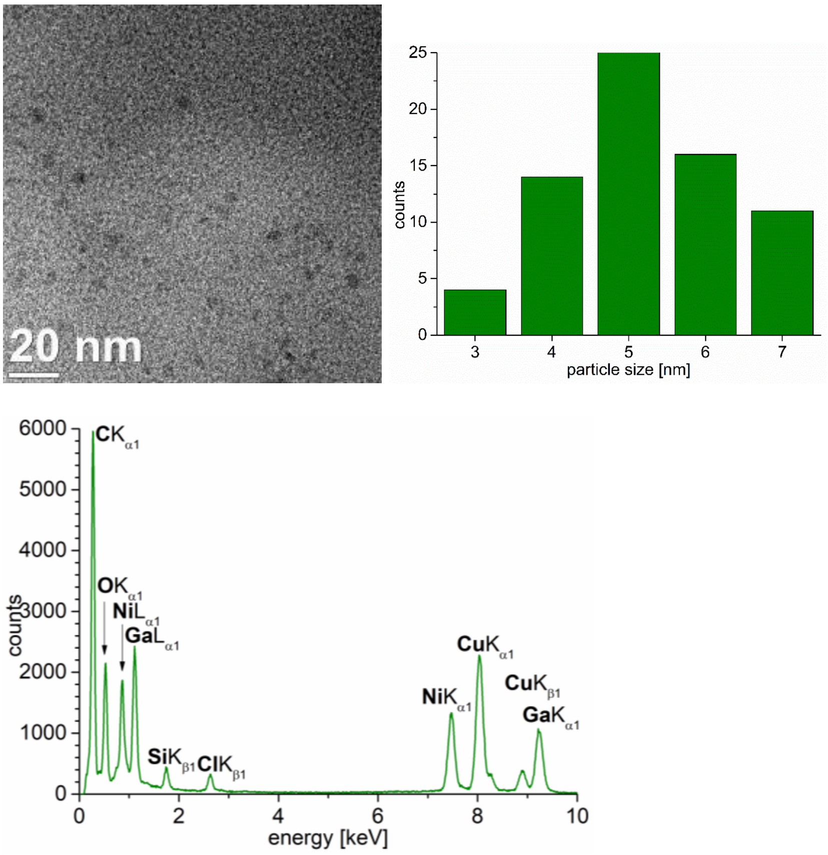

Figure S4: HR-TEM-Images and particle size distribution of the Ni/Ga-nanoparticles from 0.5 wt-% dispersion of $Ni(COD)_2$ and GaCp* in propylene carbonate (PC). 24 hours dispersion time before decomposition, 20 minutes microwave decomposition. Particle size 5 ± 1 nm (70 particles counted). EDX-Quantification from three different spots on the TEM-Grid showed equimolar ratio of nickel to gallium of 45:55 atom % (±1 atom %). No SAED-measurement possible.

Table S1: Comparison of d-spacing values (hkl-index) of experimental results (exp.) vs NiGa, Ga and Ga-rich phases. For literature d-spacing values the nine most intense reflections were chosen.

exp.	NiGa [$Pm\bar{3}m$]	Ga [$Cmce$]	Ni_3Ga_4 [$Ia\bar{3}d$]	Ni_2Ga_3 [$P\bar{3}m$]	Ni_3Ga_7 [$Im\bar{3}m$]	$NiGa_5$ [$I4/mcm$]
0.398		0.382 (020)	0.466 (211)	0.489 (001)<br>0.351 (100)	–	–
0.283	0.288 (100)	0.294 (111)	0.305 (321)<br>0.285 (400)	0.285 (101)	0.267 (310)<br>0.244 (222)	0.271 (211)
0.231		0.225 (200)	–	–	0.226 (321)	0.223 (220)<br>0.213 (114/213)
0.201	0.204 (110)	0.199 (131)	0.208 (521)<br>0.202 (440)	0.203 (110)<br>0.201 (102/012)	0.199 (330/411)	0.200 (310)<br>0.193 (204)
0.181		0.178 (221)	–	–	0.189 (420)	–
0.164	0.167 (111)		0.165 (444)	–	0.172 (422)	–
0.141	0.144 (200)	0.140 (023)	0.143 (800)	0.143 (202)	0.149 (440)<br>0.141 (600)	–
0.134	0.129 (210)		–	–	–	0.132 (404)
0.127		0.124 (331)<br>0.122 (152)	0.128 (840)	–	–	0.127 (334)<br>0.125 (217)
0.121	0.118 (211)	0.119 (223)	0.117 (844)	0.117 (300)<br>0.117 (122/21$\bar{2}$)<br>0.115 (104)	0.115 (633)	–

Table S2: EDX-quantification results from NiGa- and Ga(Ni)-nanoparticles in [BMIm][NTf_2], 1 hour and 12 hours dispersion time.

mostly large Ga(Ni)- next to some small NiGa-nanoparticles in [BMIm][NTf_2], 1 hour dispersion time					
spot number	#1	#2	#3	Ø	σ
Ni (K) [%]	28	24	30	28	2
Ga (K) [%]	72	76	70	72	2
NiGa- and Ga(Ni)-nanoparticles in [BMIm][NTf_2], 12 hour dispersion time					
spot number	#1	#2	#3	Ø	σ
Ni (K) [%]	36	38	40	38	1
Ga (K) [%]	64	62	60	62	1

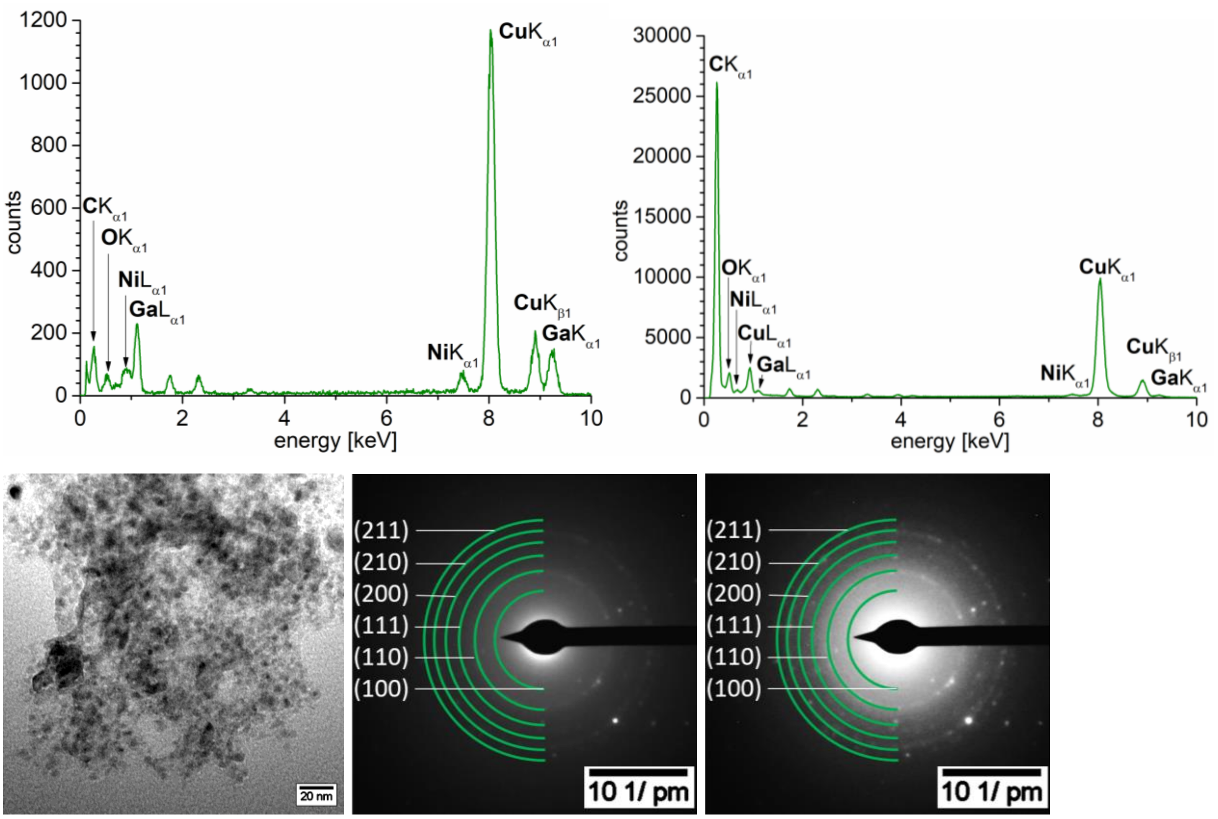

Figure S5: Top: EDX-spectra of mostly large Ga(Ni)-nanoparticles (left) and only small NiGa-nanoparticles (right) from a 0.5 wt % dispersion of $Ni(COD)_2$ and GaCp* in [BMIm][NTf_2] after 1 hour dispersion time prior to 30 min decomposition. Bottom: HR-TEM-image of NiGa-nanoparticles. SAED with indexed reflections for NiGa (green diffraction rings for space group $Pm\bar{3}m$) with two different contrasts.

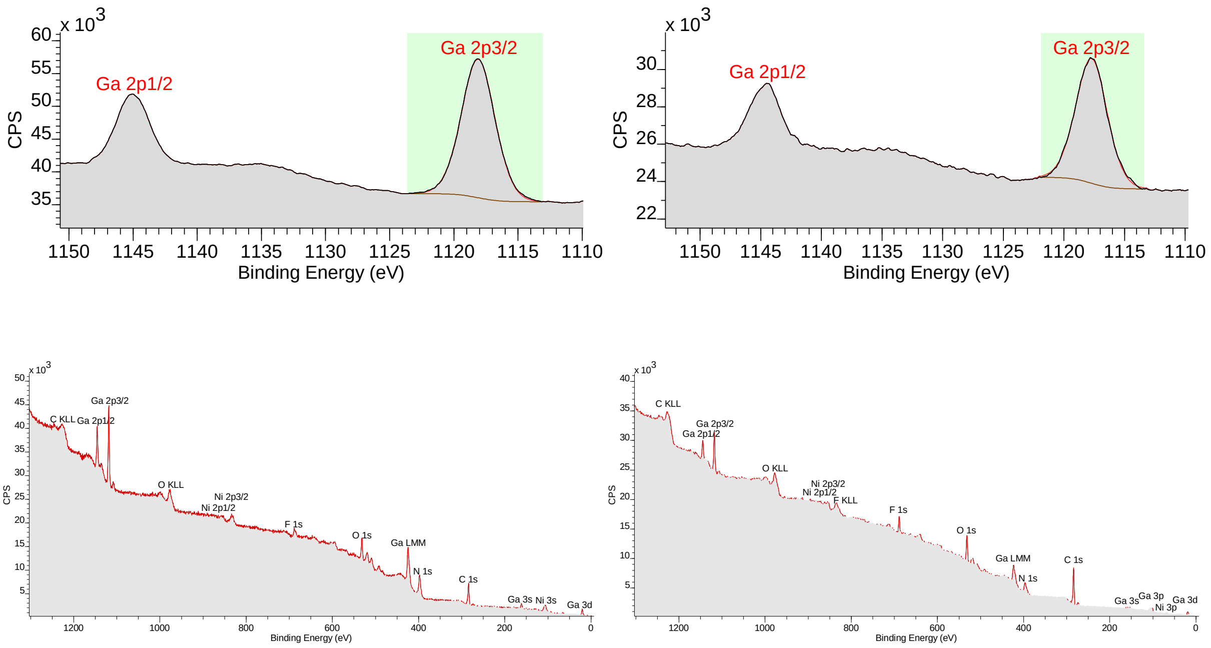

Figure S6: Top: HR-XP-spectral region of the Ga $2p_{1/2}$ and $2p_{3/2}$-orbital of NiGa/ Ga(Ni)-nanoparticles samples obtained by microwave-induced decomposition after 1 hour dispersion time (left) and 12 hours dispersion time (right). Bottom: full XP-spectra of NiGa/ Ga(Ni)-nanoparticles samples obtained by microwave-induced decomposition after 1 hour dispersion time (left) and 12 hours dispersion time (right).

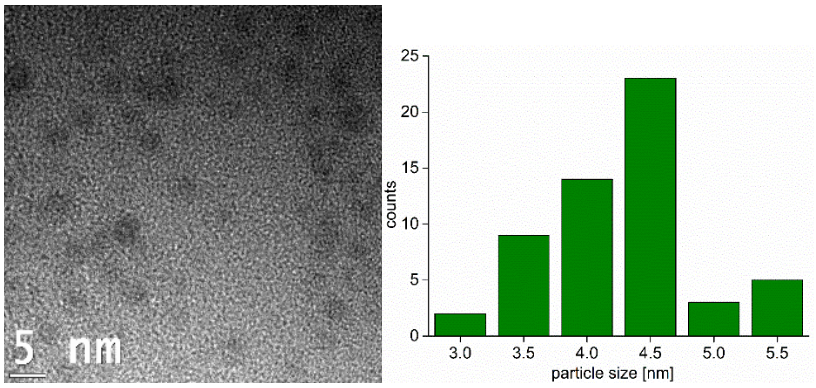

Figure S7: HR-TEM-Images and particle size distribution of the Ni-nanoparticles from 1 wt % dispersion of $Ni(COD)_2$ and GaCp* in $[EMIm][B(CN)_4]$. 30 minutes dispersion time, 30 minutes microwave decomposition. Particle size: 4 ± 1 nm (56 particles counted). EDX-Quantification from three different spots on the TEM-Grid showed only nickel. No SAED-measurement possible.

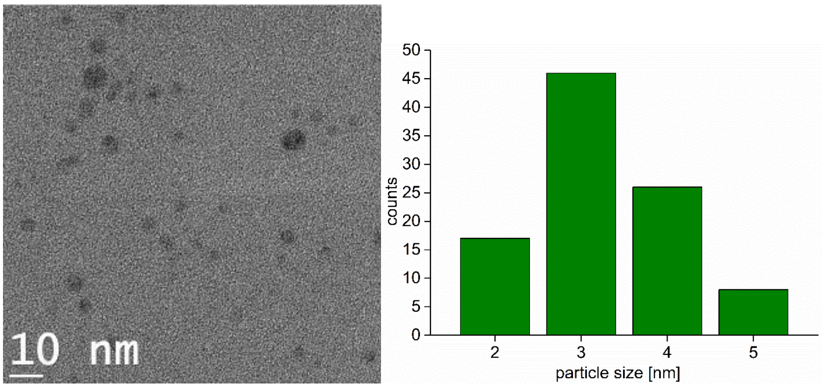

Figure S8: HR-TEM-Images and particle size distribution of the Ni/Ga-nanoparticles from 1 wt % dispersion of $Ni(COD)_2$ and GaCp* in [EMIm][$B(CN)_4$]. 24 hours dispersion time, 30 minutes microwave decomposition. Particle size: 4 ± 1 nm (97 particles counted). EDX-Quantification from different spots on the TEM-Grid showed No SAED-measurement possible.

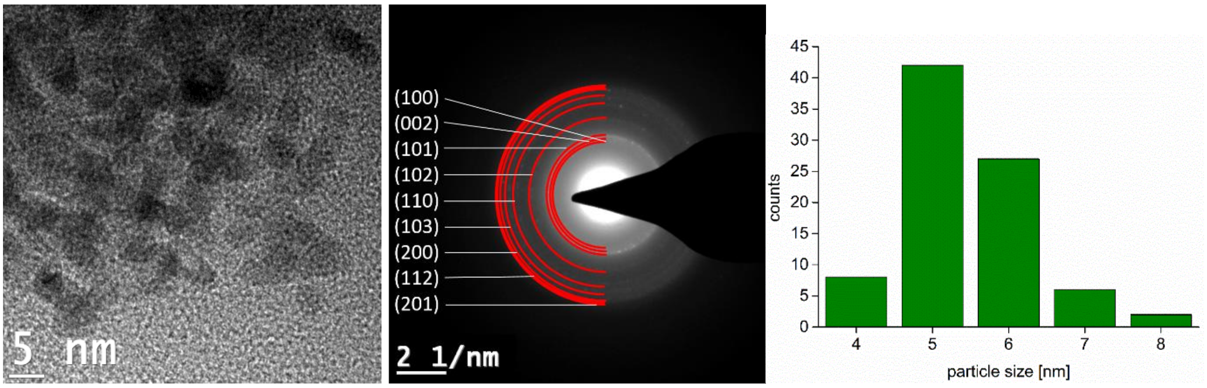

Figure S9: TEM-image and particle size distribution of 1.0 wt % Ni-nanoparticles in [EMIm][$FB(CN)_3$] from $Ni(COD)_2$ and GaCp*. 1 hour dispersion time, 30 minutes microwave decomposition. SAED with indexed reflections for Ni (space group: $P6_3/mmc$), particle size: 5 ± 1 nm (85 particles counted). EDX-Quantification from this spot showed ratio of nickel to gallium of 87:13 atom % (±1 atom %).

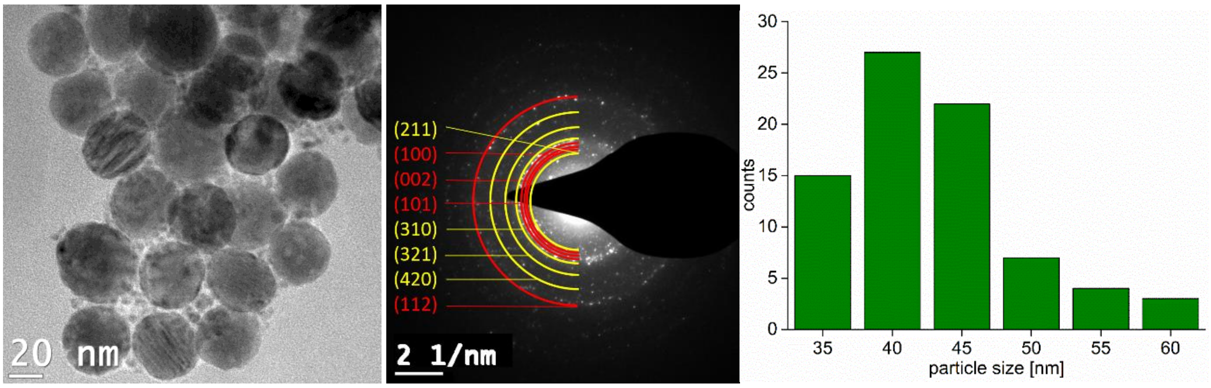

Figure S10: TEM-image and particles size distribution of 1.0 wt % Ni- and Ga(Ni)-nanoparticles in $[EMIm][FB(CN)_3]$ from $Ni(COD)_2$ and GaCp*. 1 hour dispersion time, 30 minutes microwave decomposition. SAED with indexed reflections for Ni (red diffraction rings for space group: $P6_3/mmc$) and Ga (yellow diffraction rings for space group: $I\bar{4}3d$), particle size: Ni: 5 ± 1 nm, Ga(Ni): 40 ± 5 nm. EDX-Quantification from this spot showed ratio of nickel to gallium of 49:51 atom % (±1 atom %).

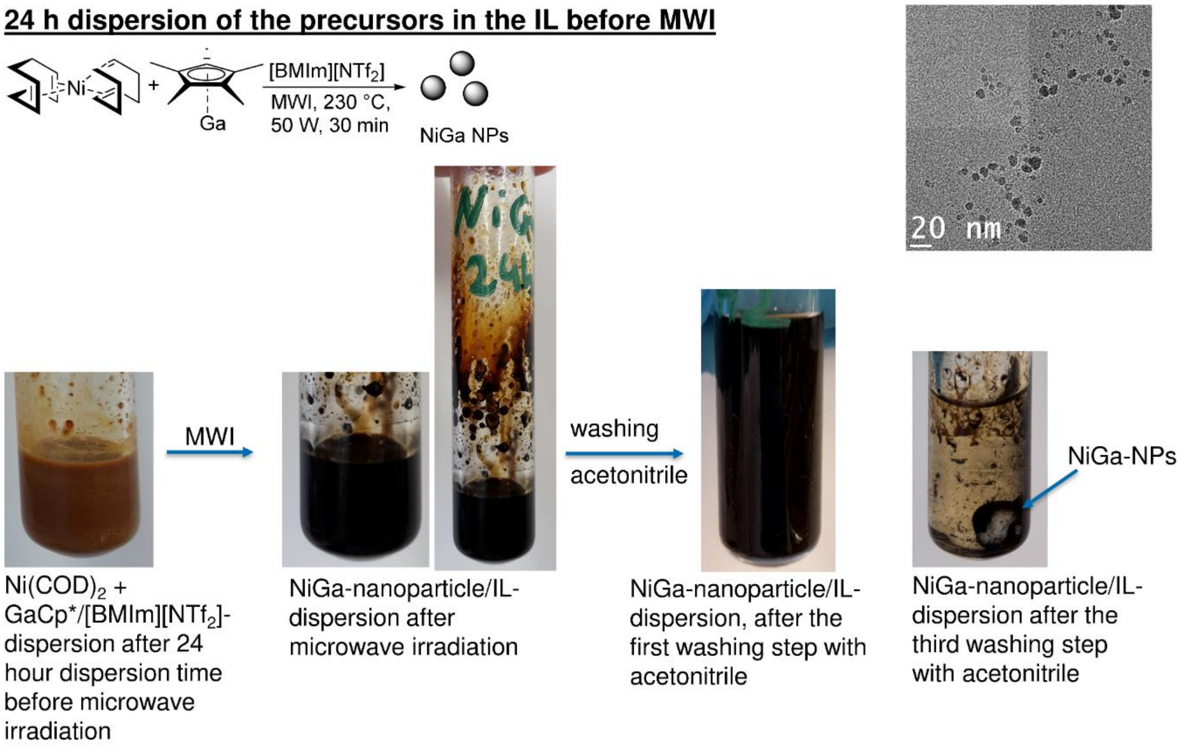

Figure S11: preparation of NiGa-nanoparticles from 24 hour dispersion time and 30 minutes decomposition time.

Table S3: Semihydrogenation of 4-octyne to 4-octene using IL-free, precipitated NiGa-nanoparticles in comparison to NiGa@[BMIm][NTf$_2$].[a]

	NiGa-nanoparticles				NiGa@[BMIm][NTf$_2$]			
	conversion [%]	selectivity 4-octene [%]	TON –	TOF [h^{-1}]	conversion [%]	selectivity 4-octene [%]	TON –	TOF [h^{-1}]
run 1	93–96	82–89	157	170	15-19	100	384	128
run 2	92–93	95–100	175	211	10	100	322	107
run 3	82–92	96–100	143	154	20	100	558	186

[a] 0.1 g NiGa@[BMIm][NTf$_2$] dispersion (1 wt % = 0.001 g in total metal, 7.8 µmol NiGa) and 2 g (2.7 mL, 18.2 mmol) of degassed dry 4-octyne (molar NiGa:substrate ratio = 1:2331) were stirred under 5 bar H_2 at 120 °C for 3 h. Runs 1–3 were carried out with the same catalyst by removing the products in high vacuum. In a typical catalytic test reaction 10 mg precipitated, IL-free NiGa-nanoparticles (77 µmol) and 1 g (1.35 mL, 9 mmol) of degassed dry 4-octyne (molar NiGa:substrate ratio = 1:115) were stirred under 5 bar H_2 at 120 °C. Runs 1–3 were carried out with the same catalyst by removing the products in high vacuum. TON per run (TON = $mol_{substrate}/mol_{catalyst}$). TOF [h^{-1}] per run (TOF = $mol_{substrate}/(mol_{catalyst}$*time)).

Table S4: Mass of $Ni(COD)_2$, GaCp* IL and PC, wt % total metal dispersion, dispersion and decomposition times in IL or PC.

IL/PC	IL/PC g (mL)	$Ni(COD)_2$ mg (mmol)	GaCp* mg (mmol)	wt % total metal dispersion	dispersion time [h]	decomp. time [min]
**[BMIm][BF$_4$]**	1.9341 (1.51)	43.1 (0.157)	31.8 (0.154)	0.995	24	10
**[BMIm][BF$_4$]**	1.2012 (0.94)	13.1 (0.048)	9.8 (0.048)	0.499	24	20
**[BMIm][NTf$_2$]**	1.2064 (0.84)	13.1 (0.048)	10 (0.049)	0.502	24	10
**[BMIm][NTf$_2$]**	0.9998 (0.70)	12.6 (0.046)	8.2 (0.040)	0.544	24	30
**[BMIm][NTf$_2$]**	1.0057 (0.70)	11.7 (0.043)	10.3 (0.050)	0.572	1	30
**[BMIm][NTf$_2$]**	1.0023 (0.70)	22.3 (0.081)	19.4 (0.094)	1.066	12	30
**PC**	1.2001 (1.00)	13.1 (0.048)	10.0 (0.0049)	0.504	24	20
**[EMIm][B(CN)$_4$]**	1.0015 (0.96)	22.4 (0.081)	18.4 (0.089)	1.0045	0.5	30
**[EMIm][B(CN)$_4$]**	0.2533 (0.24)	8.8 (0.032)	6.2 (0.030)	1.493	24	20
**[EMIm][BF(CN)$_3$]**	1.0045 (0.94)	22.5 (0.082)	17.6 (0.085)	1.025	1	30
**[EMIm][BF(CN)$_3$]**	0.3156 (0.19)	10.6 (0.039)	7.9 (0.038)	1.478	24	30
**[BMIm][NTf$_2$]**	1.0586 (0.74)	–	15.6 (0.076)	0.492	24	60

[1] H. Okamoto, *J. Phase Equilib. Diffus.* **2010**, *31*, 575–576.
